# Clinical severity in Parkinson’s disease is determined by decline in cortical compensation

**DOI:** 10.1093/brain/awad325

**Published:** 2023-09-27

**Authors:** Martin E Johansson, Ivan Toni, Roy P C Kessels, Bastiaan R Bloem, Rick C Helmich

**Affiliations:** Donders Institute for Brain, Cognition and Behaviour, Radboud University Medical Center, Centre of Expertise for Parkinson & Movement Disorders, 6525 EN Nijmegen, The Netherlands; Donders Institute for Brain, Cognition and Behaviour, Radboud University, 6525 EN Nijmegen, The Netherlands; Department of Medical Psychology, Radboud University Medical Center, 6525 GA Nijmegen, The Netherlands; Radboudumc Alzheimer Center, Radboud University Medical Center, 6525 GA Nijmegen, The Netherlands; Vincent van Gogh Institute for Psychiatry, 5803 AC Venray, The Netherlands; Donders Institute for Brain, Cognition and Behaviour, Radboud University Medical Center, Centre of Expertise for Parkinson & Movement Disorders, 6525 EN Nijmegen, The Netherlands; Donders Institute for Brain, Cognition and Behaviour, Radboud University Medical Center, Centre of Expertise for Parkinson & Movement Disorders, 6525 EN Nijmegen, The Netherlands

**Keywords:** Parkinson’s disease, action selection, fMRI, compensation, interindividual differences

## Abstract

Dopaminergic dysfunction in the basal ganglia, particularly in the posterior putamen, is often viewed as the primary pathological mechanism behind motor slowing (i.e. bradykinesia) in Parkinson’s disease. However, striatal dopamine loss fails to account for interindividual differences in motor phenotype and rate of decline, implying that the expression of motor symptoms depends on additional mechanisms, some of which may be compensatory in nature. Building on observations of increased motor-related activity in the parieto-premotor cortex of Parkinson patients, we tested the hypothesis that interindividual differences in clinical severity are determined by compensatory cortical mechanisms and not just by basal ganglia dysfunction.

Using functional MRI, we measured variability in motor- and selection-related brain activity during a visuomotor task in 353 patients with Parkinson’s disease (≤5 years disease duration) and 60 healthy controls. In this task, we manipulated action selection demand by varying the number of possible actions that individuals could choose from. Clinical variability was characterized in two ways. First, patients were categorized into three previously validated, discrete clinical subtypes that are hypothesized to reflect distinct routes of α-synuclein propagation: diffuse-malignant (*n* = 42), intermediate (*n* = 128) or mild motor-predominant (*n* = 150). Second, we used the scores of bradykinesia severity and cognitive performance across the entire sample as continuous measures.

Patients showed motor slowing (longer response times) and reduced motor-related activity in the basal ganglia compared with controls. However, basal ganglia activity did not differ between clinical subtypes and was not associated with clinical scores. This indicates a limited role for striatal dysfunction in shaping interindividual differences in clinical severity. Consistent with our hypothesis, we observed enhanced action selection-related activity in the parieto-premotor cortex of patients with a mild-motor predominant subtype, both compared to patients with a diffuse-malignant subtype and controls. Furthermore, increased parieto-premotor activity was related to lower bradykinesia severity and better cognitive performance, which points to a compensatory role.

We conclude that parieto-premotor compensation, rather than basal ganglia dysfunction, shapes interindividual variability in symptom severity in Parkinson’s disease. Future interventions may focus on maintaining and enhancing compensatory cortical mechanisms, rather than only attempting to normalize basal ganglia dysfunction.

## Teaser

Using fMRI during a visuomotor task, Johansson *et al.* show that interindividual differences in the clinical severity of Parkinson’s disease are determined by compensatory cortical mechanisms and not just by basal ganglia dysfunction. Stronger parieto-premotor activation is associated with less bradykinesia and a more benign subtype.

## Introduction

Bradykinesia is one of the cardinal motor symptoms of Parkinson’s disease. It manifests as slowness during the selection and execution of voluntary movements, worsening as task complexity increases.^1-3^ The severity of bradykinesia, which varies substantially between individuals, has been considered an outcome of dopamine depletion and basal ganglia dysfunction.^4,5^ However, motor symptoms may also be shaped by compensatory cortical processes.^2,3,6-11^ It remains unclear to what extent such compensatory processes, along with interindividual differences in their efficacy, contribute to clinical heterogeneity in Parkinson’s disease, over and above basal ganglia dysfunction. We tested this by analysing data from a cohort of patients (Personalized Parkinson Project^12^; *n* = 353) who performed an action selection task designed to activate compensatory cerebral mechanisms implicated in motor control whilst undergoing functional MRI.

In Parkinson’s disease, progressive degeneration of dopaminergic neurons in the substantia nigra leads to dopamine depletion in the striatum, resulting in dysfunctional basal ganglia output and impaired motor performance.^5,13-17^ These motor impairments become particularly pronounced in situations where patients are required to make voluntary choices between multiple competing movement options,^18-22^ potentially as a result of increased cognitive load.^3,23,24^ However, striatal dopamine depletion occurs gradually over several years prior to the onset of bradykinesia.^8,15,25-29^ Furthermore, motor symptoms progress despite the fact that the motor region of the striatum (posterior putamen) is almost entirely depleted of dopamine 4 years after diagnosis.^25^ Additionally, several studies have failed to demonstrate an association between changes in motor symptom severity and striatal dopamine levels over time^15,30-33^ (although see Parkinson Study Group^34^). These observations strongly imply that basal ganglia dysfunction is not the sole mechanism underlying bradykinesia and action selection deficits in Parkinson’s disease.

Over recent decades, several studies have suggested that action selection is partially maintained in Parkinson’s disease by compensatory cerebral processes.^7^ Cerebral compensation has been conceptualized as a performance-enhancing recruitment of neural resources that enable individuals to meet moderately high task demands despite deficits in the neural mechanisms that typically support task performance.^11,35,36^ Cerebral compensation can manifest in at least two forms: upregulated activation of mechanisms already dedicated to task performance, or selective recruitment of mechanisms that are typically dedicated to other processes.^37,38^ Such compensatory mechanisms are thought to be instantiated during the long preclinical phase of Parkinson’s disease, which may last for several years,^39,40^ to stabilize behavioural performance as basal ganglia dysfunction gradually worsens.^6,8,10,41,42^ However, with disease progression, the degree of basal ganglia dysfunction will eventually exceed the capacities of compensatory mechanisms, leading to the appearance and subsequent worsening of motor deficits.^10,43,44^ Importantly, the efficacy of these compensatory mechanisms likely differs between individuals owing to idiosyncrasies in patterns of pathology (e.g. focal versus diffuse propagation of α-synucleinopathy) and may therefore contribute to the clinical heterogeneity that characterizes Parkinson’s disease.^45,46^

Demonstrating compensation with neuroimaging is not straightforward, since increased brain activity during a task may reflect either recruitment of compensatory resources or reduced efficiency of processes that support task performance.^35,47^ Two basic functional criteria for establishing compensation have been suggested. First, it should be clear what is being compensated for, such as basal ganglia dysfunction in the case of Parkinson’s disease.^5^ Second, compensatory brain activity should have beneficial effects on behavioural performance.^11^ Furthermore, recruitment of compensatory mechanisms is expected particularly when task demands are higher, i.e. when pathological deficits would otherwise lead to impaired performance. More generally, demonstrating compensation requires statistical power adequate to detect biologically plausible effects linking neural compensation and behavioural performance in Parkinson’s disease.^48,49^ Recent meta-analyses of functional MRI studies have shown that patients with Parkinson’s disease have increased motor-related activation in parieto-premotor regions that are implicated in healthy motor control.^16,17,50^ This increase in activity is suggestive of compensatory upregulation, e.g. reflecting enhanced reliance on goal-directed control during motor execution.^7^ However, most studies were unable to verify this possibility due to sample sizes inadequate to quantify brain-behaviour correlations. Other studies have focused on presymptomatic carriers of gene mutations associated with a high risk of developing Parkinson’s disease, who showed intact behavioural performance in combination with increased premotor activity relative to healthy controls during action selection.^51,52^ This selection-related increase in premotor activation decreases as motor symptoms worsen after the onset of Parkinson’s disease, indicating that it may reflect compensatory mechanisms in the presymptomatic stage.^53^ Finally, we and others have shown that patients with Parkinson’s disease rely more heavily on the extrastriate visual cortex during motor imagery of their most affected hand,^54^ and that disruption of this region with transcranial magnetic stimulation impaired motor imagery in Parkinson’s disease patients but not in healthy controls.^55^ This imagery-related extrastriate visual cortex activity might be compensatory, but a relationship with actual motor behaviour has not yet been established.

Here, we tested the hypothesis that the clinical heterogeneity in Parkinson’s disease depends on compensatory upregulation of parieto-premotor function, over and above basal ganglia dysfunction. We used a motor task in combination with functional MRI to assess motor- and selection-related brain activity in early-to-moderate Parkinson’s disease patients.^56^ We verified that Parkinson’s disease is associated with reduced activity in the basal ganglia by comparing task-related activity in patients against healthy controls. We also tested for normalizing effects of dopamine replacement therapy on motor network dysfunction. The novelty of this study concerns the neural mechanisms underlying clinical heterogeneity in Parkinson’s disease. First, we compared clinical subtypes (mild-motor predominant, intermediate and diffuse-malignant) that were defined based on motor symptoms, cognitive performance, REM sleep behaviour disorder and autonomic dysfunction.^57^ Second, we quantified the relationship between interindividual variability in brain activity and the clinically rated severity of bradykinesia as well as a composite measure of global cognitive performance. Our findings show that enhanced parieto-premotor activity is related to a more benign subtype of Parkinson’s disease, less severe bradykinesia and better cognitive performance, suggesting that it may be compensating for a basal ganglia deficit.

## Materials and methods

### Participants

Data from 367 patients diagnosed with idiopathic Parkinson’s disease and 60 healthy controls were retrieved from the Personalized Parkinson Project database in March 2022. The Personalized Parkinson Project is an ongoing single-centre longitudinal cohort study taking place at Radboud University Medical Center (Nijmegen, the Netherlands; ClinicalTrials.gov identifier: NCT03364894 and NCT05169827).^12^ All patients underwent sequential motor symptom assessments OFF (i.e. 12 h since the last dose of dopaminergic medication) and ON dopaminergic medication with the Movement Disorders Society Unified Parkinson Disease Rating Scale part III (MDS-UPDRS III).^58^ MRI measurements were acquired in the ON-medicated state. Fifty-six patients returned for identical MRI measurements OFF medication. Half of these 56 patients were assessed within 3 months after their first ON-state measurement. The other half were assessed within 3 months prior to a 2-year follow-up visit, to reduce potential learning effects and even out the distribution of disease durations in the OFF-medicated group. Healthy controls were matched to the OFF-medicated group with respect to age, sex and handedness. Written informed consent was obtained for all participants in accordance with the Declaration of Helsinki. The study was approved by a medical ethical committee (METC Oost-Nederland, formerly CMO Arnhem-Nijmegen; #2016-2934 and #2018-4785). See the Supplementary material for detailed information on inclusion and exclusion criteria. During baseline assessments, diagnoses of eight patients were re-evaluated to either a form of atypical parkinsonism (*n* = 2) or other (*n* = 6). Diagnosis re-evaluations at 2-year follow-up confirmed that an additional six patients did not have Parkinson’s disease (three multiple system atrophy, two progressive supranuclear palsy and one indeterminate). All patients with a verified non-Parkinson’s disease diagnosis at either baseline or follow-up were excluded from further analysis, resulting in a total sample size of 353 ON-medicated patients (of whom 55 also had an OFF-medication assessment) and 60 controls. All healthy controls had complete data. Numbers of missing and excluded data points from patients can be found in the Supplementary material. Demographic information can be found in Table 1.

**Table 1:** Demographic information and clinical characteristics

Variable	Control	PD-OFF	PD-ON	MMP	IM	DM	Undefined
Sample size	60	54	353	150	128	42	33
Age	60.0 (9.6)	61.1 (8.5)	62.5 (8.6)	61.4 (8.9)	63.1 (8.5)	65.5 (6.9)	61.7 (8.8)
Sex (F/M)	27/33	22/32	129/224	67/83	46/82	8/34	8/25
Years of education	16.2 (3.3)	17.1 (4.1)	17.2 (4.1)	17.3 (4.3)	17.3 (4.3)	16.5 (4.3)	16.4 (3.7)
Dominant hand (L/R)	5/55	7/47	56/297	25/125	21/107	8/34	2/31
Responding hand (L/R)	29/31	28/24	167/178	66/81	66/60	22/19	13/18
Responding hand is dominant (N/Y)	26/34	27/25	159/186	63/84	62/64	19/22	15/16
Disease duration (months)	NA	34.2 (15.6)	34.2 (17.3)	32.7 (16.6)	34.9 (17.9)	34.8 (17.7)	37.4 (17.3)
Most affected side (L/R/Bi)	NA	27/23/5	173/163/17	66/79/5	65/57/6	17/21/4	15/16/2
Hoehn and Yahr-stage	NA	11/37/5/1	40/265/44/3	22/117/11/0	13/103/11/1	0/26/14/2	5/19/8/0
Medication use (N/Y)	NA	0/54	17/335	11/139	3/125	0/42	3/29
LEDD	NA	501.5 (220.3)	545.2 (319.8)	467.8 (256.5)	573.8 (334.1)	624.1 (353.7)	715.4 (405.9)
MoCA	27.6 (1.9)	27.1 (1.8)	26.7 (2.6)	27.2 (2.0)	26.7 (2.5)	26.0 (3.5)	25.9 (3.4)
Cognitive composite	NA	0.2 (0.5)	0.0 (0.6)	0.3 (0.4)	−0.2 (0.6)	−0.4 (0.8)	−0.3 (0.6)
MDS-UPDRS II	NA	7.7 (5.0)	8.4 (5.6)	5.9 (4.1)	8.8 (4.8)	15.3 (6.2)	9.5 (6.8)
**MDS-UPDRS III: OFF-state assessment**							
Total	NA	33.6 (13.8)	33.7 (13.0)	29.6 (10.9)	33.3 (12.3)	47.8 (10.5)	36.4 (15.6)
Bradykinesia	NA	16.2 (7.7)	16.7 (7.5)	14.4 (6.5)	16.7 (7.0)	24.6 (6.4)	17.5 (8.3)
Rigidity	NA	7.0 (3.0)	6.6 (3.3)	6.1 (3.2)	6.6 (3.3)	8.7 (3.0)	6.4 (3.3)
PIGD	NA	2.1 (1.4)	2.0 (1.4)	1.6 (1.0)	1.9 (1.1)	3.9 (1.7)	2.2 (1.4)
Tremor	NA	5.8 (5.1)	6.1 (5.2)	5.9 (5.0)	5.9 (5.2)	7.2 (5.2)	7.6 (5.6)
**MDS-UPDRS III: ON-state assessment**							
Total	NA	28.0 (13.3)	28.7 (12.5)	25.4 (10.6)	29.2 (13.0)	38.7 (10.8)	28.3 (14.0)
Bradykinesia	NA	13.7 (7.2)	14.4 (7.2)	12.6 (6.5)	14.5 (7.3)	20.0 (6.2)	13.7 (7.6)
Rigidity	NA	6.0 (3.3)	6.0 (3.3)	5.5 (3.1)	6.0 (3.5)	7.7 (3.1)	5.5 (3.3)
PIGD	NA	1.6 (1.3)	1.6 (1.2)	1.2 (0.9)	1.6 (1.1)	2.6 (1.6)	1.7 (1.2)
Tremor	NA	4.6 (3.5)	5.0 (4.5)	4.6 (4.1)	5.3 (5.0)	5.3 (4.4)	5.2 (3.1)

Values are presented as mean (SD) unless otherwise stated. Bi = bilateral; DM = diffuse-malignant; F = female; FWEc = family-wise error cluster; IM = intermediate; L = left; LEDD = levodopa equivalent daily dose; M = male; MDS-UPDRS = Movement Disorders Society Unified Parkinson Disease Rating Scale; MMP = mild-motor predominant; MoCA = Montreal Cognitive Assessment; N = no; PD-OFF = patients scanned in the OFF-medicated state; PD-ON = patients scanned in the ON-medicated state; PIGD = postural instability and gait disorder; R = right; Y = yes.

### Action selection task

#### Task instructions

**Figure 1:**
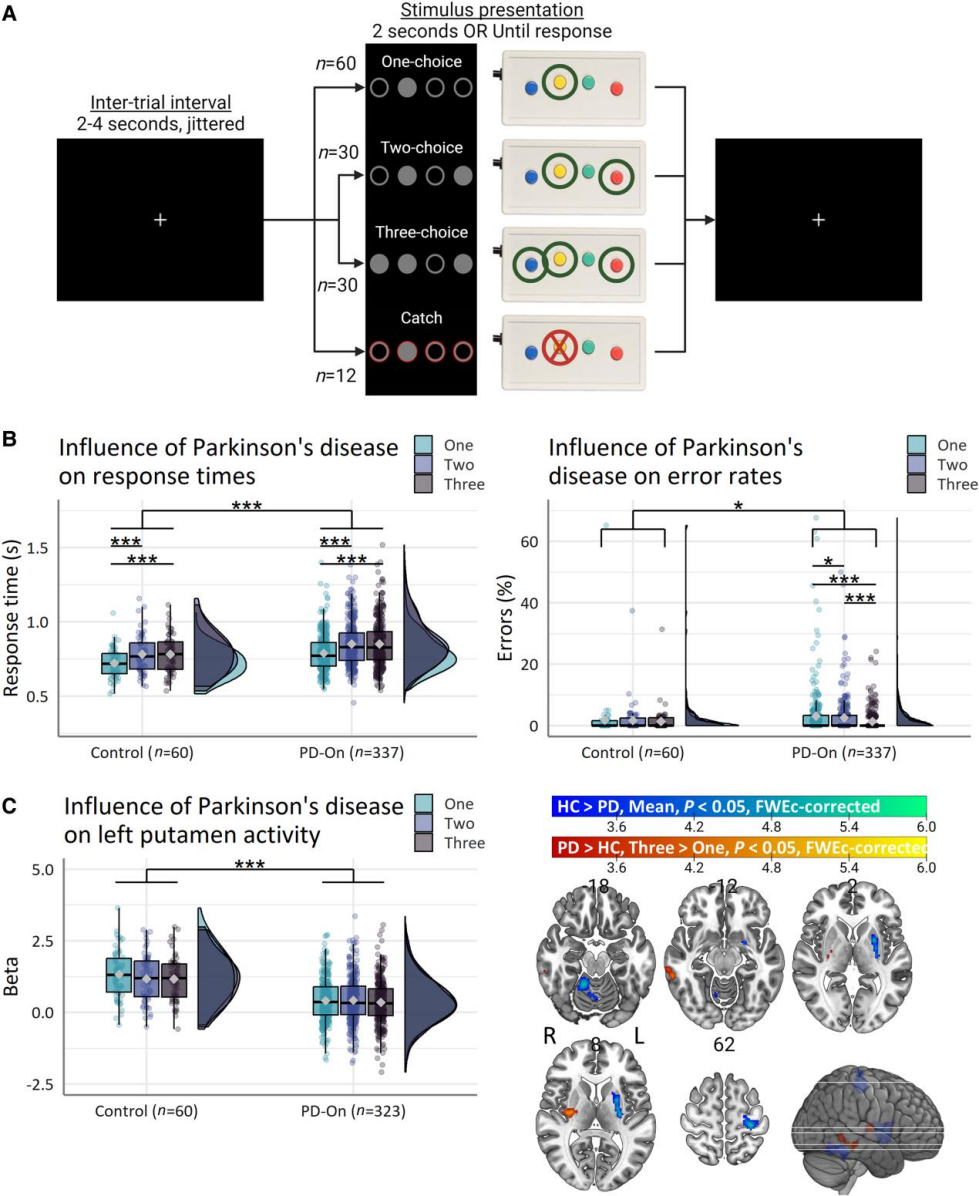
**Action selection task and Parkinson-related deficits**. (**A**) Participants respond to highlighted circles with one out of four response buttons. When multiple circles are highlighted, participants are instructed to select one response button. Action selection demand is parametrically manipulated by varying the number of highlighted circles that are presented. During catch trials, participants are instructed to withhold a response. No feedback was given to indicate the correctness of responses. (**B**) Parkinson’s disease leads to general deficits in task performance. (**C**) Parkinson’s disease leads to reduced motor-related activity in a network of core motor regions and reduced selection-related deactivation in the middle temporal gyrus. Box plots show the first and third quartiles (boxes), median (horizontal line), mean (diamond) and 1.5 × interquartile range (whiskers). Brain images show *t*-values of significant clusters. FWEc = Family-wise error cluster; HC = healthy control; PD = Parkinson’s disease; PD-On = ON-medicated Parkinson’s disease; SE = standard error. *P < 0.05, ***P < 0.001.

Participants performed an action selection task whilst undergoing mixed block/event-related functional MRI (Fig. 1A).^59-61^ This task was specifically designed to elicit compensatory mechanisms that may contribute to preserving motor (bradykinesia) and cognitive function. This was achieved by assessing action selection deficits resulting from Parkinson’s disease under varying levels of task difficulty.^21,22^ Participants were instructed to respond to highlighted cues with a single button press as quickly and as accurately as possible and to try to make equal use of all response options. The number of highlighted cues varied between one and three. If multiple cues were highlighted, participants were instructed to choose and respond to one cue only, which imposed greater demands on action selection capabilities. Behavioural performance was assessed during low (one-choice), moderate (two-choice) and high (three-choice) action selection demand. See Supplementary material for a more detailed account of the task.

#### Measurements of behavioural performance

Response times, errors and misses were aggregated within participants by trial condition (one-choice, two-choice, three-choice) and block (one, two, three), resulting in nine values per participant for each measurement. Response times were aggregated by taking the median after excluding misses, incorrect responses and response times below 300 ms. Errors were similarly aggregated by taking the number of incorrect trials divided by the total number of correct and incorrect trials, excluding misses. Misses were aggregated by taking the number of misses divided by the total number of trials. In trials where multiple choices were possible, participants sometimes had the option to repeat their previous response or switch to a new response. Based on such trials, two additional metrics (response variability and switching) were calculated to characterize the use of stereotyped response strategies.^53,62^ First, for each of the four response options, the number of button presses were calculated. Response variability (i.e. the degree to which all available response options were used) was subsequently characterized as a coefficient of variation, calculated by taking the standard deviation of button presses across response options divided by the mean. Larger coefficients of variation indicate that button presses are not uniformly distributed (i.e. that there was a bias towards one or more response options). Second, response switching (i.e. the degree to which the same response was repeated over consecutive trials) was characterized as the ratio of switches relative to the total number of switches and repeats. Smaller ratios indicate that response repetitions are more likely than switches.

### Subtyping

We used a recently developed^57^ and previously validated^46,63^ clinical subtype classification to parse clinical heterogeneity. The classification used in the present study has been described in detail in a previous publication where we investigated clinical differences between subtypes in the larger Personalized Parkinson Project cohort.^46^ In short, patients were classified based on motor symptoms, cognitive function, REM movement sleep behaviour disorder and autonomic function (Supplementary material). Classification resulted in three distinct subtypes: diffuse-malignant (*n* = 42), intermediate (*n* = 128) and mild-motor predominant (*n* = 150). Thirty-three patients could not be classified due to missing data. In short, the diffuse-malignant subtype showed relatively severe motor symptoms, impairment in more clinical domains and faster progression in comparison to the mild-motor predominant subtype.^46^

### Clinical measurements

Action selection involves motor performance under cognitive load. Clinical scores of motor symptoms and cognitive performance were therefore used to test for associations with task-related performance and brain activity. In the motor domain, bradykinesia was specifically selected as a symptom-of-interest owing to its previously established relationship with action selection.^18-22^ Bradykinesia severity was defined as the sum of 11 MDS-UPDRS III items (assessed in the OFF-medicated state) targeting bradykinesia (4–9 and 14).^64,65^ Cognitive performance was defined as the same composite score that was used for subtype classification, which was based on extensive neuropsychological assessment. This composite score was computed by taking the mean across age-, education- and sex-adjusted *z*-scores from tests in six cognitive domains (visuospatial perception, executive function, semantic fluency, processing speed, working memory and episodic memory; Supplementary material).

### Image acquisition and preprocessing

All scans were acquired using a Siemens MAGNETOM Prisma 3T (Siemens) equipped with a 32-channel head coil. T1-weighted anatomical images were acquired using a magnetization-prepared rapid gradient-echo sequence [repetition time (TR)/echo time (TE)/inversion time (TI) = 2000/2/880 ms; flip angle = 8°; voxel size = 1.0 × 1.0 × 1.0 mm; slices = 192; field of view (FOV) = 256 mm; scanning time = 5 min). T2*-weighted functional images were acquired during the performance of the action selection task using a multi-band sequence (TR/TE = 1000/34 ms; acceleration factor = 6; acquisition mode = interleaved; flip angle = 60°; voxel-size = 2.0 × 2.0 × 2.0 mm; slices = 72; FOV = 210 mm; scanning time = 9–10 min).

Preprocessing of functional MRI data was performed using a standardized pipeline in fmriprep (v20.2.1).^66^ In short, functional images were motion- and slice time-corrected and normalized to MNI152Lin6Asym-space. Lastly, corrected and normalized images were spatially smoothed with Gaussian kernel of 6 mm at full-width half-maximum. See Supplementary material for detailed information.

### First-level analysis

SPM12 (https://www.fil.ion.ucl.ac.uk/spm/software/spm12) was used to carry out all first-level and group-level analyses. Task regressors were generated for one-, two- and three-choice conditions by convolving cue onsets with a canonical haemodynamic response function. Cue duration was defined as the average response time across choice conditions. Time derivatives were included for each condition together with parametric regressors for response time and their first-order derivatives. Additional regressors were generated for catch trials and incorrect responses. Confound time series were included in the first-level model to correct for anatomical and motion-related sources of noise (Supplementary material). Task-related activity for each choice condition was defined by contrasting each separate task regressor against an implicit baseline (one-choice > 0; two-choice > 0; three-choice > 0; catch > 0). The resulting contrast images were used as inputs for group comparisons. Additional contrasts were formed to encode motor-related [(one-choice, two-choice, three-choice) > 0] and selection-related (two-choice > one-choice; three-choice > one-choice) activity (see Supplementary Fig. 1 for mean activation associated with each contrast of interest). These contrasts were used to assess associations with bradykinesia severity and cognitive performance. Contrast images of all participants who responded with the left side were flipped horizontally to ensure that the most-affected side was consistent across patients.

### Statistical analysis

#### Behavioural performance

The influences of disease status (between-subjects factor Group: ON-medicated patients versus controls), dopaminergic medication (within-subjects factor Group: ON- versus OFF-medicated states) and subtype (between-subjects factor Group: mild-motor predominant versus intermediate versus diffuse-malignant; ON-medicated state only) were assessed with linear mixed-effects models for log-transformed response times and with weighted binomial logistic mixed-effects models for error and miss rates using the lme4-package^67^ in R 4.2.1 (R Core Team). Each model included fixed effects for Group and Choice (within-subjects factor Choice: one-choice, two-choice, three-choice) as well as their interaction. Repeated measures were accounted for with by-subject random intercepts. Block was included as a fixed effect to account for task habituation. Associations between behavioural performance and clinical severity were assessed within patients using the same model formula, with the exception that the factor of Group was removed and a term was added for Symptom severity, defined as the bradykinesia subscore of the MDS-UPDRS III (assessed in the OFF-medicated state) or the cognitive composite score. Models were fitted using a restricted maximum likelihood approach. *P*-values for fixed effects were derived through type III analyses of deviance using Wald *χ*^2^ tests. Response variability and switching were analysed using one-way analyses of covariance (ANCOVAs), with Group as a between-subjects factor. Clinical associations were assessed within patients using similar mixed-effects modelling as above. All behavioural analyses included age, sex, years of education and dominance of the responding hand as covariates of non-interest. Two-tailed *post hoc t*-tests were performed on estimated marginal means. Participants with less than 25% correct responses on one-choice trials, averaged across blocks, were excluded from further analysis. In analyses of response variability and switching, additional outliers scoring above or below three standard deviations from the mean were excluded.

#### Brain activity

Group comparisons of task-related brain activity closely followed the group comparisons that were conducted for behavioural performance. Three separate repeated-measures ANCOVAs, implemented using the full factorial design option in SPM12, were used to carry out voxel-wise tests for effects of disease status (ON-medicated patients versus controls), medication (ON- versus OFF-medicated states) and subtype (mild-motor predominant versus intermediate versus diffuse-malignant; patients assessed in the ON-medicated state) on brain activity. Estimates of brain activity for each task condition (one-choice, two-choice, three-choice, catch) were used as inputs. Contrasts were set up to compare motor- and selection-related activity between groups (see Supplementary material for average task-related effects). The comparison between subtypes was followed by a *post hoc* comparison between each subtype and controls.

The relationship between brain activity and clinical severity was investigated with voxel-wise one-way ANCOVAs where bradykinesia severity and cognitive performance were simultaneously considered as regressors of interest and fitted to first-level contrasts of motor- and selection-related activity. This enabled the assessment of the contributions of bradykinesia and cognitive performance independently of each other. Separate correlation analyses for each clinical domain (Supplementary material) and a conjunction analysis were conducted *post hoc* to assess between-domain cluster overlap. Mean framewise displacement, age, sex, years of education and responding hand dominance were included as covariates of non-interest in all analyses of brain activity. Here, years of education controlled for individual differences in functional reserve^68,69^ whereas hand dominance controlled for differential extent of bilateral recruitment between dominant versus non-dominant hand responders.^70,71^ Cluster-based thresholding, with a cluster-forming threshold of *Z* = 3.1, was used to correct for family-wise error at *P* < 0.05.^72^ Anatomical labels and functional subdivisions of significant clusters were derived from the JuBrain Anatomy Toolbox (v3.0)^73^ and Glasser atlas,^74^ respectively.

Two control analyses were carried out. First, interindividual differences in compensatory capacity may be influenced by underlying differences in reserve, which can be defined as a pre-morbid accumulation of neural resources that reduces susceptibility to the effects of pathological decline.^11,69,75^ In the analyses above, four reserve proxies were already accounted for (i.e. age, sex, years of education and dominant side onset).^76^ However, reserve may be influenced by additional factors. This was explored in between-subtype comparisons and brain-clinical correlations that adjusted for four additional reserve proxies: body-mass index, smoking history, physical activity and non-motor burden (Supplementary material).^1,68,76^ Second, between-group differences in cortical compensation may result from underlying differences in brain structure. Voxel-based morphometry was therefore used to compare gray matter volume in regions that showed significant group differences in task-related activity (Supplementary material).

## Results

### The influence of disease status

#### Behavioural performance

Response times were ‘longer’ in Parkinson patients than controls [Fig. 1B; main effect of Group *χ*^2^(1) = 11.2, *P* < 0.001, *η^2^_p_* = 0.03; patient > control; log-ratio = 1.07, standard error (SE) = 0.02, *t*-ratio(391) = 3.3, *P* = 0.001], and they increased with action selection demand [main effect of Choice *χ^2^*(2) = 79.7, *P* < 0.001, *η^2^_p_* = 0.07; two-choice > one-choice; log-ratio = 1.08, SE = 0.006, *t*-ratio(3170) = 14.0, *P* < 0.001; three-choice > one-choice; log-ratio = 1.08, SE = 0.006, *t*-ratio(3170) = 13.5, *P* < 0.001]. Patients were not disproportionally slower for multi-choice trials than controls (no Group × Choice interaction, *P* = 0.86).

Error rates were higher in Parkinson patients than controls specifically during single-choice versus multi-choice trials compared with controls [Fig. 1B; Group × Choice *χ^2^*(2) = 6.4, *P* = 0.041; one-choice > three-choice, patient > control; odds ratio (OR) = 2.0, SE = 0.55, *Z*-ratio = 2.5, *P* = 0.035].

Miss rates were ‘higher’ in Parkinson patients than controls [main effect of Group *χ^2^*(1) = 10.0, *P* = 0.002; patient > control; OR = 3.5, SE = 1.4, *Z*-ratio = 3.2, *P* = 0.001]. There was no effect of Choice (*P* = 0.42).

There was no effect of Group on response variability (Supplementary Fig. 2A; *P* = 0.09) or switching (Supplementary Fig. 3A; *P* = 0.24).

#### Brain activity

##### Motor-related activity

Patients with Parkinson’s disease showed (reduced) motor-related activity in the left putamen, left precentral gyrus (area 4) and right cerebellum (IV–V) compared with controls (Table 2 and Fig. 1B; control > patient, mean > baseline).

**Table 2:** Voxel-wise group comparisons

Anatomical label (% cluster volume)	Area	*P*-value (FWEc-corrected)	Cluster extent (voxels)	Max *t*-value	MNI: *x*, *y*, *z*
**Influence of Parkinson’s disease**					
Control > PD-ON, Mean > Baseline					
L putamen (88%)	–	<0.001	433	5.6	−27,−15,6
L precentral gyrus (68%)	M1	<0.001	416	5.4	−31,−27,62
R cerebellum (71%)	IV-V	<0.001	313	5.6	17,−47,−20
PD-ON > Control, Two > One					
R putamen (45%)	–	0.017	120	4.7	31,−13,8
PD-ON > Control, Three > One					
R middle temporal gyrus (82%)	TE1p	0.032	185	5.1	61,−37,−14
**Influence of subtype**					
Mild-motor predominant > Diffuse-malignant, Mean > Baseline					
R postcentral gyrus (87%)	S1	0.010	116	3.9	51,−31,56
Mild-motor predominant > Diffuse-malignant, Three > One					
R middle frontal gyrus (72%)	i6-8	<0.001	421	4.6	35,17,52
R inferior parietal lobule (61%)	PFm	<0.001	220	4.2	47,−55,38
R superior parietal lobule (96%)	7Pm	0.002	150	4.3	5,−59,52
L superior parietal lobule (87%)	7Am	0.035	98	4.4	−11,−67,64
Intermediate > Diffuse-malignant, Three > One					
L middle frontal gyrus (68%)	6a	0.029	94	4.6	−21,3,56
***Post hoc* comparison between subtypes and controls**					
Mild-motor predominant > Control, Three > One					
R inferior parietal lobule (48%)	PFm	<0.001	384	5.4	51,−63,46
R middle temporal gyrus (88%)	TE1p	0.002	151	4.3	61,−37,−14
R superior frontal gyrus (72%)	9a	0.004	134	4.1	23,56,30
R middle frontal gyrus (70%)	8Av	0.016	102	4.4	27,11,40
Intermediate > Control, Two > One					
R putamen (42%)	–	0.002	149	5.6	31,−13,8
Control > Diffuse-malignant, Three > One					
L middle frontal gyrus (83%)	6a	0.009	114	4.3	−25,5,60

Anatomical labels were derived from the Anatomy Toolbox v3.0. Area labels were derived from the Glasser atlas. FWEc = family-wise error cluster; L = left; MNI = Montreal Neurological Institute; PD-ON = Patients scanned in the ON-medicated state; R = right.

##### Selection-related activity

**Figure 2:**
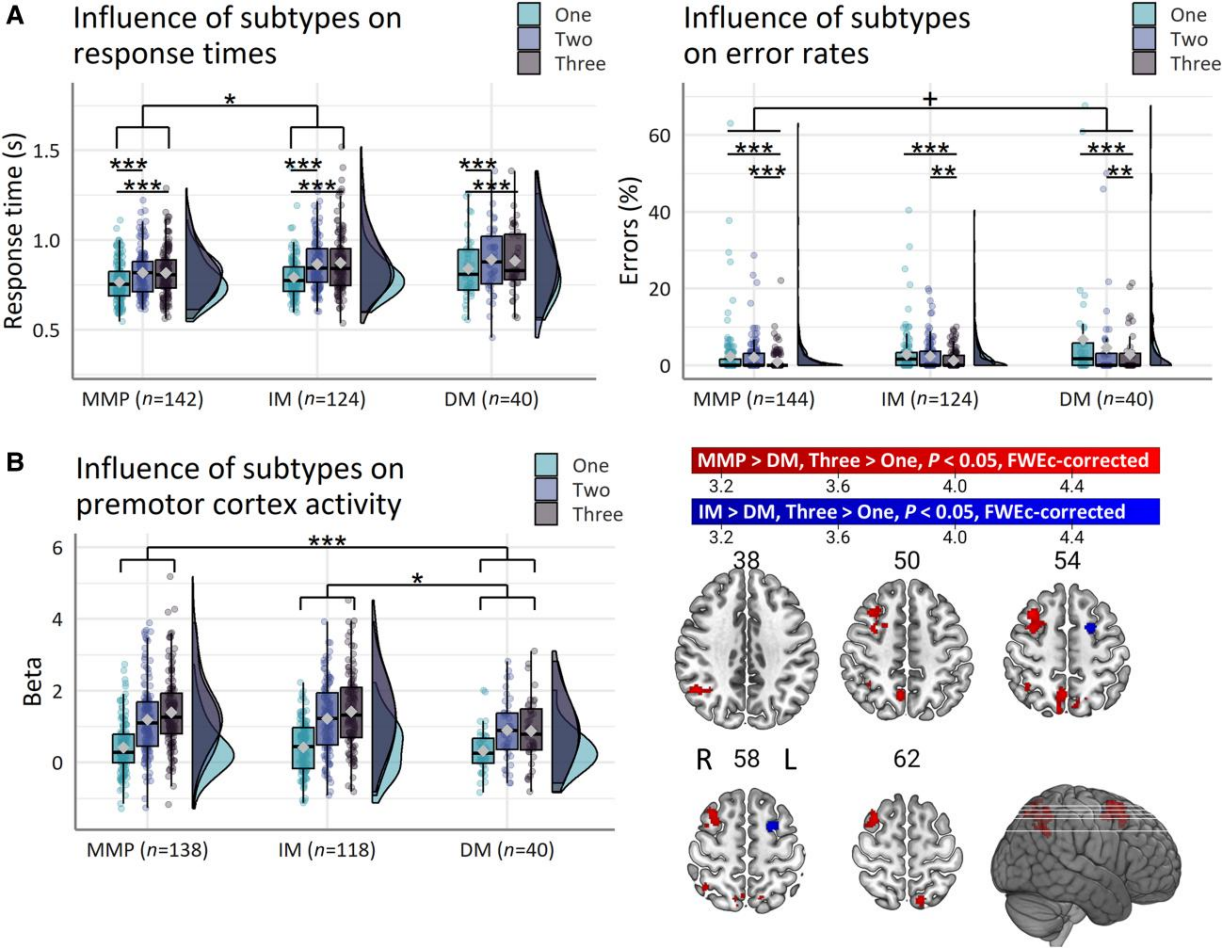
**Evidence for parieto-premotor compensation**. (**A**) Greater selection-related increase in response times in intermediate compared to mild-motor predominant patients. (**B**) Reduced motor-related activity in diffuse-malignant compared to mild-motor predominant and intermediate patients. Box plots show the first and third quartiles (boxes), median (horizontal line), mean ( diamond) and 1.5 × interquartile range (whiskers). Brain images show *t*-values of significant clusters. DM = diffuse-malignant; FWEc = family-wise error cluster; IM = intermediate; L = left; MMP = mild-motor predominant; R = right. ^+^*P* < 0.09, *P < 0.05, **P < 0.01; ***P < 0.001.

Patients with Parkinson’s disease showed differential activity in the right putamen at moderate demand compared with controls (Table 2 and Fig. 1B; patient > control, two-choice > one-choice), which was driven by a relative reduction in putamen activity during one-choice trials for patients versus controls (Supplementary Fig. 4A). Furthermore, patients showed increased activity in the right middle temporal gyrus (TE1p) at high demand compared with controls (Table 2 and Fig. 2B; patient > control, three-choice > one-choice), which was driven by opposing directions of selection-related effects between groups (Supplementary Fig. 4A).

### The influence of clinical subtype

#### Behavioural performance

The influence of action selection demand on response times differed between subtypes [Fig. 2A; Group × Choice *χ^2^*(4) = 11.9, *P* = 0.018, *η^2^_p_* = 0.005]. The difference in response times between high and low action selection demand was ‘increased’ for intermediate compared with mild-motor predominant patients [intermediate > mild-motor predominant, three-choice > one-choice; log-ratio = 1.028, SE = 0.01, *t*-ratio(2456) = 2.8, *P* = 0.032] but not for diffuse-malignant compared with mild-motor predominant patients (*P* = 0.96).

Error rate increased as a function of action selection demand [Fig. 2A; main effect of Choice *χ^2^*(2) = 34.5, *P* < 0.001; one-choice > two-choice (OR = 1.3, SE = 0.11, *Z*-ratio = 2.5, *P* = 0.031), one-choice > three-choice (OR = 2.6, SE = 0.29, *Z*-ratio = 8.6, *P* < 0.001), two-choice > three-choice (OR = 2.1, SE = 0.26, *Z*-ratio = 5.8, *P* < 0.001)]. There was a trend towards increased error rates for diffuse-malignant compared to mild-motor predominant patients [main effect of Group *χ^2^*(2) = 5.7, *P* = 0.058; diffuse-malignant > mild-motor predominant (OR = 1.8, SE = 0.54, *P* = 0.089)].

There was no effect of Group on miss rates (*P* = 0.14), response variability (Supplementary Fig. 2A; *P* = 0.64) or switching (Supplementary Fig. 3A; *P* = 0.24).

#### Brain activity

##### Motor-related activity

Patients with a mild-motor predominant subtype had ‘increased’ motor-related activity in the right postcentral gyrus (area 2) compared to patients with a diffuse-malignant subtype (Table 2 and Fig. 2B; mild-motor predominant > diffuse-malignant, mean > baseline). There were no differences in basal ganglia activity between subtypes (Supplementary Fig. 5).

##### Selection-related activity

Patients with a mild-motor predominant subtype had ‘increased’ selection-related activity in the right middle frontal gyrus (i6-8), right inferior parietal lobule (PFm), right superior parietal lobule (7Pm) and left superior parietal lobule (7Am) compared to patients with a diffuse-malignant subtype (Table 2 and Fig. 2C; mild-motor predominant > diffuse-malignant, three-choice > one-choice). Furthermore, patients with an intermediate subtype also had ‘increased’ selection-related activity in the left middle frontal gyrus (6a) compared with the diffuse-malignant subtype (Fig. 2C; intermediate > diffuse-malignant, three-choice > one-choice). These results remained significant after accounting for additional measures of reserve (Supplementary material), suggesting that a more benign clinical phenotype is associated with ‘higher’ selection-related activation in a network that involves premotor, inferior parietal and superior parietal cortex.

**Figure 3:**
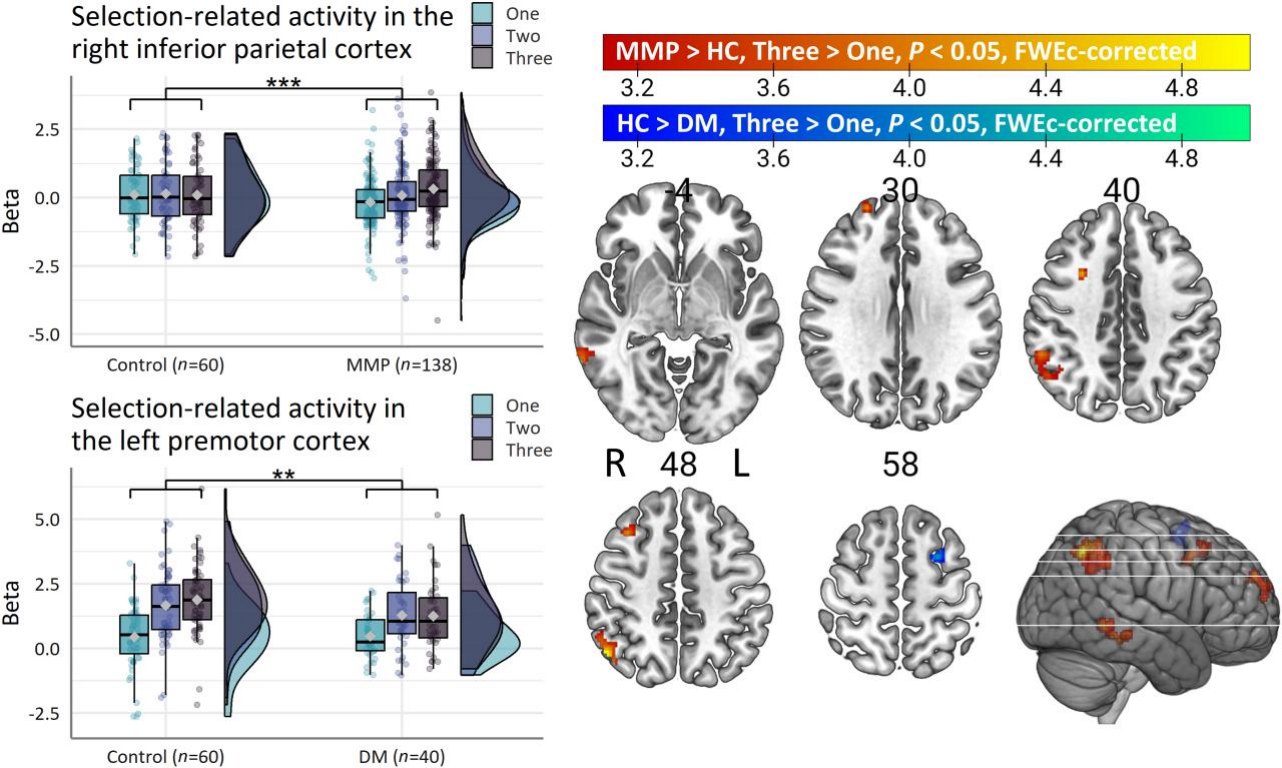
***Post hoc* comparisons between subtypes and controls**. Reduced selection-related activity in diffuse-malignant compared to mild-motor predominant and intermediate patients (MMP > HC, Three > One). In comparison to controls, mild-motor predominant patients show increased selection-related parieto-premotor activity whereas diffuse-malignant patients show the opposite (HC > DM, Three > One). Box plots show the first and third quartiles (boxes), median (horizontal line), mean (diamond) and 1.5 × interquartile range (whiskers). Brain images show *t*-values of significant clusters. DM = diffuse-malignant; FWEc = family-wise error cluster; HC = healthy control; L = left; MMP = mild-motor predominant; R = right. **P < 0.01, ***P < 0.001.

*Post hoc* comparisons of selection-related activity between subtypes and controls were carried out to assess the clinical relevance of the results above. Patients with a mild-motor predominant subtype had ‘increased’ selection-related activity in the right inferior parietal lobule (PFm), right middle temporal gyrus (TE1p), right superior frontal gyrus (9a) and right middle frontal gyrus (8Av) compared with controls (Fig. 3; mild-motor predominant > control, three-choice > one-choice). In contrast, patients with a diffuse-malignant subtype had ‘decreased’ selection-related activity in the left middle frontal gyrus (6a) compared with controls (Fig. 3; control > mild-motor predominant, three-choice > one-choice). Patients with an intermediate subtype had differential selection-related activity in the right putamen (intermediate > control, two-choice > one-choice), which was driven by ‘reduced’ putamen activity in patients versus controls for the one-choice condition (Supplementary Fig. 4B).

### Associations with clinical severity

#### Behavioural performance

Bradykinesia severity was inversely related to cognitive performance [*r* = −0.28, *t*(330) = 5.2, *P* < 0.001].

**Figure 4:**
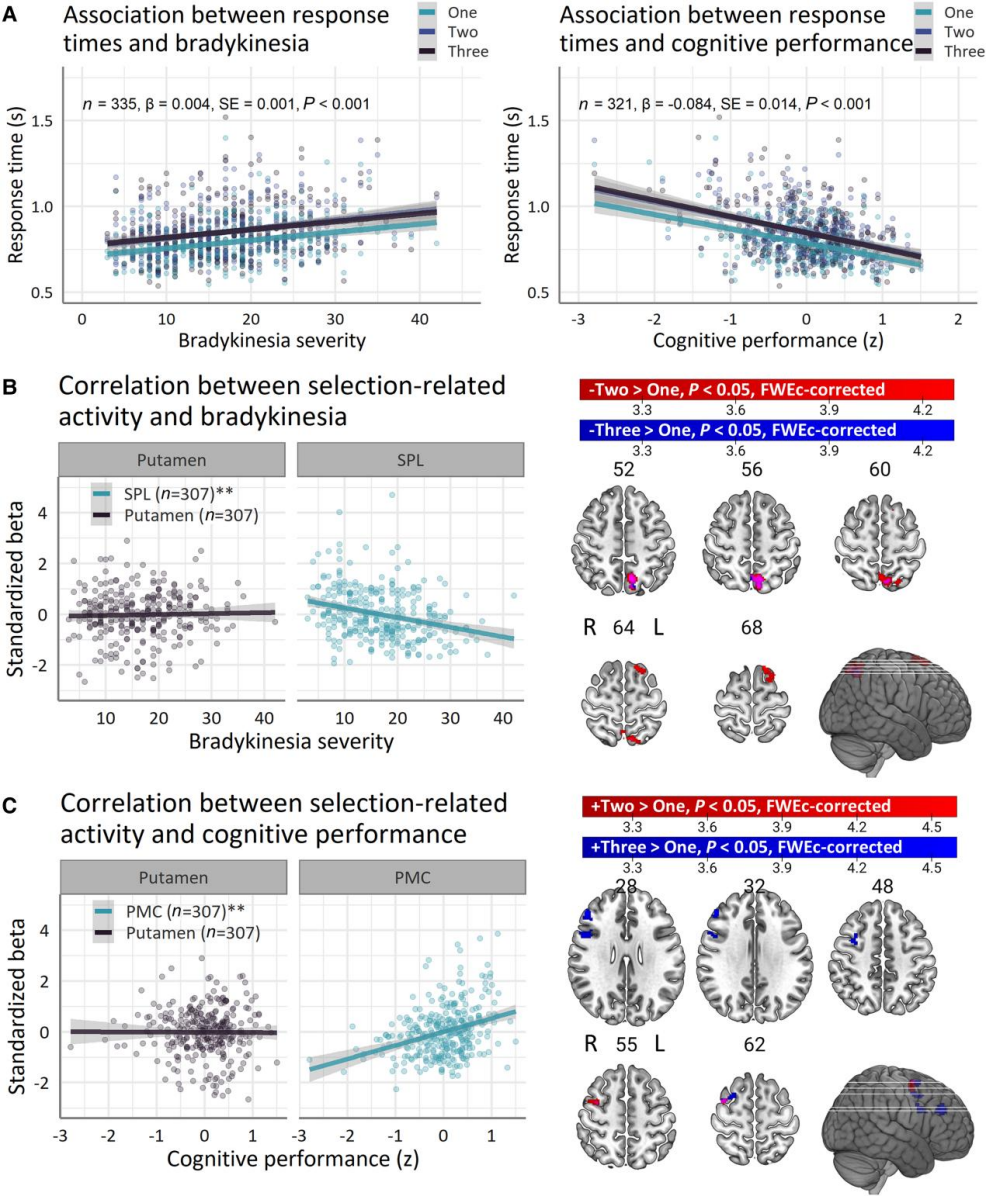
**Associations with specific clinical domains**. (**A**) Task performance predicts bradykinesia severity (*left*) and cognitive performance (*right*). (**B**) Associations with bradykinesia severity. (**C**) Associations with cognitive performance. Line-plots show linear associations (solid line) and standard errors (outline). Brain images show *t*-values of significant clusters. Bradykinesia severity was defined as a subscore of the Movement Disorders Society Unified Parkinson Disease Rating Scale part III (MDS-UPDRS part III). Cognitive performance was defined as a composite score of clinical assessments across multiple cognitive domains. FWEc = family-wise error cluster; L = left; PMC = premotor cortex; SE = standard error; SPL = superior parietal lobule; R = right; ± = positive/negative correlation. **P < 0.01, ***P < 0.001.

Response times ‘increased’ as a function of bradykinesia severity [Fig. 4A; main effect of Severity *χ^2^*(1) = 14.3, *P* < 0.001, *η^2^_p_* = 0.04] and ‘decreased’ as a function of cognitive performance [Fig. 4A; main effect of Severity *χ^2^*(1) = 34.1, *P* < 0.001, *η^2^_p_* = 0.11]. There were no interactions between Severity and Choice (both *P* > 0.3).

The influence of action selection demand on error rates depended on bradykinesia severity [Severity × Choice *χ^2^*(2) = 11.2, *P* = 0.003] and cognitive performance [Severity × Choice *χ^2^*(2) = 13.4, *P* = 0.001]. Patients with more severe bradykinesia had ‘increased’ error rates [one-choice > three-choice; *β* = 0.042, SE = 0.013, *Z*-ratio = 3.12, *P* = 0.005], whereas patients with better cognitive performance had ‘decreased’ error rates [one-choice > three-choice; *β* = −0.470, SE = 0.141, *Z*-ratio = 3.35, *P* = 0.002] during low versus high action selection demands.

Patients with more severe bradykinesia had ‘increased’ miss rates [*χ^2^*(1) = 10.1, *P* = 0.001, OR = 1.059], whereas patients with better cognitive performance had ‘decreased’ miss rates [*χ^2^*(1) = 41.0, *P* < 0.001, OR = 0.276]. There were no interactions between Severity and Choice (both *P* < 0.14).

Patients with better cognitive performance had ‘decreased’ button press variability [Supplementary Fig. 2B; *F*(1) = 6.6, *P* = 0.011, *η^2^_p_* = 0.04] and ‘increased’ switch rates [Supplementary Fig. 3B; *F*(1) = 7.4, *P* = 0.007, *η^2^_p_* = 0.05]. There were no associations between bradykinesia and response variability (Supplementary Fig. 2B; *P* = 0.15) or switching (Supplementary Fig. 3B; *P* = 0.43).

#### Brain activity

##### Motor-related activity

There were no associations with motor-related activity.

##### Selection-related activity

At moderate demands, lower bradykinesia severity was associated with ‘greater’ selection-related activity in the left superior parietal lobule (7Am) and superior frontal gyrus (6ma; Table 3 and Fig. 4B; negative correlation, two-choice > one-choice), whereas better cognitive performance was associated with ‘greater’ selection-related activity in the right middle frontal gyrus (i6-8; Table 3 and Fig. 4C; positive correlation, two-choice > one-choice). At high demand, associations with bradykinesia became more constrained to the superior parietal cortex, while associations with cognitive performance extended to additional prefrontal regions. Specifically, lower bradykinesia severity was associated with ‘greater’ selection-related activity in the left superior parietal lobule (7Am; Table 3 and Fig. 3B; negative correlation, three-choice > one-choice). Better cognitive performance was associated with ‘greater’ selection-related activity in the right middle frontal gyrus (areas i6-8 and p9-46v) and right inferior frontal gyrus (6r; Table 3 and Fig. 4C; positive correlation, three-choice > one-choice) and with ‘less’ selection-related activity in the left cuneus (V2; negative correlation, three-choice > one-choice). A *post hoc* conjunction analysis revealed no brain regions where selection-related activity correlated with bradykinesia severity and cognitive performance. When analysed separately, bradykinesia severity (Supplementary Fig. 7A) and cognitive performance (Supplementary Fig. 7B) showed negative and positive correlations, respectively, with a wider network of parieto-premotor regions as a function of increasing action selection-demand (Supplementary material and Supplementary Tables 1 and 2).

**Table 3:** Voxel-wise clinical correlations

Anatomical label (% cluster volume)	Area	*P*-value (FWEc-corrected)	Cluster extent (voxels)	Max *t*-value	MNI: *x*, *y*, *z*
**Two > One**					
Bradykinesia, negative correlation					
L superior parietal lobule (83%)	7Am	<0.001	381	4.9	−9,−61,52
L superior frontal gyrus (76%)	6ma	0.002	152	4.4	−15,19,66
Cognitive performance, positive correlation					
R middle frontal gyrus (97%)	i6-8	0.016	103	4.3	35,−1,58
**Three > One**					
Bradykinesia, negative					
L superior parietal lobule (92%)	7Am	0.001	166	4.3	−9,−63,54
Cognitive performance, positive correlation					
R middle frontal gyrus (52%)	i6-8	0.003	147	4.0	31,9,46
R middle frontal gyrus (54%)	p9-46v	0.013	114	4.6	47,36,28
R inferior frontal gyrus (62%)	6r	0.029	96	3.9	45,9,28
Cognitive performance, negative correlation					
L cuneus (70%)	V2	0.022	102	4.5	−9,−94,20

Anatomical labels were derived from the Anatomy Toolbox v3.0. Area labels were derived from the Glasser atlas. FWEc = family-wise error cluster; L = left; MNI = Montreal Neurological Institute; R = right.

### The influence of medication

Motor symptom severity decreased following dopaminergic medication [MDS-UPDRS-III total *χ^2^*(1) = 221.1, *P* < 0.001, *η^2^_p_* = 0.40; bradykinesia *χ^2^*(1) = 127.3, *P* < 0.001, *η^2^_p_* = 0.29]. Dopaminergic medication led to ‘decreased’ miss rates during low versus high action selection demand [Group × Choice *χ^2^*(1) = 8.0, *P* = 0.019; Parkinson’s disease-ON > Parkinson’s disease-OFF, Three > One (OR = 0.35, SE = 0.14, *Z*-ratio = 2.72, *P* = 0.018)]. There were no additional effects of dopaminergic medication on task performance or brain activity (Supplementary material and Supplementary Fig. 8).

## Discussion

We investigated the cerebral mechanisms underlying clinical heterogeneity in Parkinson's disease in a cohort of early-to-moderately affected^56^ patients (*n* = 353) and healthy controls (*n* = 60). By leveraging clinical subtyping and brain-symptom associations, we showed that lower symptom severity was consistently associated with higher activation in superior parietal and premotor cortex, particularly during high demands on action selection. In contrast, we found no evidence for a relationship between symptom severity and basal ganglia activity, which was reduced in all clinical subtypes, independently of symptom severity, compared to controls. These findings support the hypothesis that interindividual variability in symptom severity in Parkinson’s disease may be determined by compensatory cortical processes rather than basal ganglia dysfunction.

### Action selection deficits are associated with basal ganglia dysfunction and clinical severity

We used an action selection paradigm to investigate cerebral mechanisms underlying abnormal motor control in Parkinson’s disease, which implicates both motor symptoms (e.g. bradykinesia) and cognitive deficits (e.g. decision-making), and which was designed to elicit cortical compensatory mechanisms. Consistent with previous neuroimaging and behavioural studies, we found that Parkinson’s disease was associated with reduced motor-related activity in a core network of sensorimotor regions (putamen, primary motor cortex and cerebellum) accompanied by general slowing of motor performance, lower accuracy and more misses.^16,17,77,78^ Furthermore, larger deficits in each of the three behavioural indices were associated with higher severity of bradykinesia and lower cognitive performance. In combination, these findings suggest that the task was sensitive to both action selection deficits and basal ganglia dysfunction.

This sensitivity was built into the task by virtue of two design improvements over previous action selection tasks.^21,53,79^ First, we varied the number of response options parametrically on a trial-by-trial basis, using a mixed block/event-related design with a jittered inter-stimulus interval.^59-61^ We also explicitly instructed participants to make equal use of all response options. This reduced predictability in the task, motivating participants to generate responses based on a process of selection rather than a predefined strategy, such as responding with the same finger on each trial.^7^ Indeed, we found no between-group differences in response variability or switching. In combination with the behavioural deficits described above, this suggests that Parkinson’s disease patients and controls utilized similar behavioural strategies, although the task was relatively more demanding for patients, especially at higher levels of disease severity. The parametric variation of response options additionally enabled us to show that compensatory cerebral mechanisms are recruited primarily when action selection demands increase (see later). Second, patients were asked to respond with their most affected hand, which enhances sensitivity to Parkinson’s disease-related deficits and excludes a potential difference in action selection capacity between the right and left sides.

### Evidence for a compensatory role of parieto-premotor cortex

This study provides evidence that the parieto-premotor cortex may support a compensatory role in Parkinson’s disease. Conceptually, compensatory cerebral alterations involve a performance-enhancing recruitment of neural resources that maintain behaviour during relatively demanding movements despite deficits in the neural mechanisms that typically support their performance.^10,11,41^ This can be distinguished from the related concept of neural reserve, which manifests as a trait-like property that confers beneficial effects across clinical domains in a task-independent manner.^11,35^ Accordingly, motor impairments are expected to emerge when dysfunction exceeds the capacities of compensatory mechanisms that support motor performance, such as increased reliance on sensory cueing^80^ or goal-directed control.^7^ This may explain why motor symptoms differentially worsen during voluntary movements that require a selection between multiple actions.^18-21^

**Figure 5:**
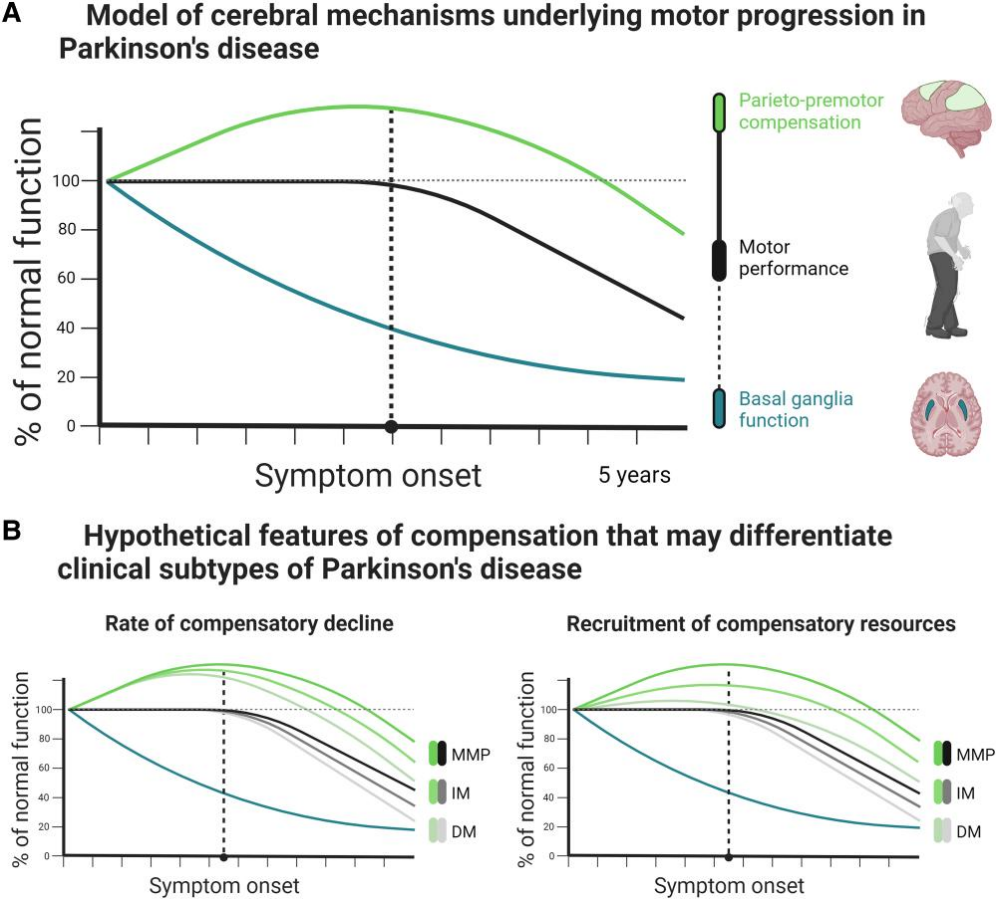
**Model of the relationship between changes in brain activity and motor symptom progression**. (**A**) Parieto-premotor activity is enhanced to compensate for basal ganglia dysfunction during the presymptomatic phase of Parkinson’s disease. Eventually, parieto-premotor compensation begins to decline, leading to the emergence of motor impairments caused by basal ganglia dysfunction. According to this model, decline in motor performance depends on loss of cortical compensation rather than progressive basal ganglia dysfunction. (**B**) Subtypes may be differentiated by the rate at which cortical compensation declines, the degree to which cortical resources can be recruited for compensatory purposes, or a combination of the two. DM = diffuse-malignant; IM = intermediate; MMP = mild-motor predominant.

Our findings fit with a compensatory role for the parieto-premotor cortex in Parkinson’s disease, for the three following reasons. First, we demonstrate that Parkinson’s disease is associated with decreased basal ganglia activity and impaired action selection performance. This demonstrates the presence of dysfunction in one brain region, which would in turn call for compensation elsewhere in the nervous system. Second, we report stronger selection-related upregulation of parieto-premotor cortex activity in Parkinson patients with the most benign clinical subtype, consistent with the expected dependency between compensation and behavioural demand.^11^ Specifically, compared to healthy controls, mild-motor predominant patients showed ‘increased’ parieto-premotor activity, whereas diffuse-malignant patients showed ‘reduced’ premotor activity. The lack of between-group differences in response variability and switching further suggests that these effects were not caused by differences in the ability to perform the task. Third, we provide evidence that upregulated parieto-premotor activity is not just a by-product of inefficient cerebral processing: across the entire cohort, a stronger enhancement of parieto-premotor activity was independently associated with both lower bradykinesia severity and better cognitive performance. In combination, these findings suggest that enhanced parieto-premotor function may confer beneficial effects on motor performance by partially compensating for deficits in basal ganglia function,^11,37^ which fits with the recent reconceptualization of bradykinesia as a consequence of large-scale network dysfunction.^2,9,81-83^ Importantly, these findings remained intact even when accounting for multiple proxies of reserve (Supplementary material). Accordingly, we predict that the worsening of motor symptoms is primarily driven by decline in parieto-premotor compensation (Fig. 5A). We are currently addressing this hypothesis in an upcoming study where we extend the methodology of the present study to a longitudinal setting.

### Neural mechanisms underlying focal and diffuse clinical subtypes

Subtypes of Parkinson’s disease have been linked to functional and structural alterations that may contribute to differences in clinical phenotype, symptom severity and progression.^84^ For example, the diffuse-malignant subtype, which is characterized by relatively severe motor and non-motor symptoms,^46^ is associated with increased excitability and reduced plasticity in the primary motor cortex,^85^ wide-spread disruptions in structural connectivity,^86^ and more extensive atrophy at both cortical and subcortical levels.^57,86^ These alterations may reflect decline in neural reserves that enable the recruitment of compensatory mechanisms during behaviourally demanding tasks.^11^ We add to these findings by showing that the diffuse-malignant subtype is characterized by more extensive cortical dysfunction, but not by a differential reduction in basal ganglia dysfunction. More specifically, we show that the diffuse-malignant subtype is characterized by deficits in the compensatory upregulation of parieto-premotor activity during action selection, which remains intact in the mild-motor predominant subtype. Weaker compensation in diffuse-malignant patients may result from deficits in the ability to recruit compensatory resources (Fig. 5B, left), faster decline in compensatory function (Fig. 5B, right), or both.

Variability in compensatory function likely depends on subtype-specific differences in pathological mechanisms, such as the spread of α-synucleinopathy.^87^ α-synucleinopathy may begin either in the CNS, spreading towards the peripheral nervous system (‘brain-first’), or the other way around (‘body-first’),^88^ resulting in distinct clinical phenotypes that resemble the subtypes that we utilized in this study.^89^ That is, a brain-first form of α-synucleinopathy (which overlaps with the clinical ‘mild motor-predominant’ subtype) has been linked to younger disease onset and motor symptoms that are confined to single effectors, likely as a result of somatotopically dependent retrograde nigral degeneration.^90^ The body-first form, on the other hand, has been associated with older disease onset, diffuse symptomatology involving multiple clinical domains and rapid clinical progression, which overlaps with the clinical ‘diffuse-malignant’ subtype. These phenotypic differences suggest that a brain-first type of pathology leads to focal deficits in brain function that are restricted to cortico-striatal loops whereas a body-first type leads to diffuse deficits that involve more widespread cortical networks.^89^ Hence, the diffuse-malignant subtype may be characterized by weaker parieto-premotor compensation as a result of diffuse cortical α-synucleinopathy, which effectively constrains the neural resources that patients with this subtype are able to recruit for compensatory purposes.

### Mechanisms underlying parieto-premotor compensation

While the mechanisms that enable the parieto-premotor cortex to compensate for basal ganglia dysfunction cannot be directly inferred from this study, we suggest that they may involve non-motor territories of the basal ganglia (anterior striatum and the caudate nucleus)^37,38,91^ and motor control pathways that bypass the basal ganglia.^92^ A recent theory holds that sensorimotor regions specify and select between competing motor responses through a process of evidence accumulation towards a decision boundary.^93,94^ This process is dynamically influenced by urgency signals originating from the basal ganglia that determine the vigour of responses in a contextually dependent manner.^95,96^ In Parkinson’s disease, urgency signalling is disrupted by dopamine depletion, thereby increasing the amount of evidence that sensorimotor regions must accumulate to activate motor patterns, leading to loss of movement vigour (slowness and inability to initiate movements). However, sensorimotor regions receive additional biasing inputs that may be relied on to compensate for basal ganglia dysfunction. For example, cognitive and affective territories of the basal ganglia that are relatively spared from dopamine depletion in earlier stages of Parkinson’s disease may become increasingly involved in action selection.^7,37,97^ Furthermore, movements can be triggered via pathways that bypass the basal ganglia,^92^ which is consistent with a wealth of evidence suggesting that patients can rely on sensory input, such as visual cues,^80^ to compensate for movement deficits. We therefore speculate that an upregulation of parieto-premotor function in Parkinson’s disease may reflect increased processing of inputs from non-motor territories of the basal ganglia as well as other cortical regions that enable sensorimotor regions to exceed decision boundaries and select actions despite dysfunctional urgency signalling.

Hemispheric lateralization of activity may offer potential insight into the compensatory nature of selection-related parietal cortex activity.^98,99^ For example, lower bradykinesia severity was preferentially associated with activity in the left parietal cortex, which has been implicated in predictive motor control. Speculatively, enhanced left parietal cortex function may enable patients to more efficiently adapt movement patterns to counteract perturbations caused by underlying pathological dysfunction.^99^

### Strengths, limitations and interpretational issues

The primary strength of this study is a large sample size combined with in-depth clinical phenotyping, which enabled us to identify sources of clinical heterogeneity and quantity relationships with brain activity derived from a brief task specifically designed to elicit compensation. However, some limitations require consideration.

We did not observe previously demonstrated differential effects of action selection on response times in patients with Parkinson’s disease compared to healthy controls.^18-21^ Instead, we found that Parkinson’s disease was associated with slower response times across levels of action selection demand, suggesting a general deficit in action selection performance. In contrast with previous cohorts, ours had a relatively short disease duration (maximally 5 years), raising the possibility that deficits resulting from increased action selection demand mainly arise in later stages of Parkinson’s disease, potentially as a result of decline in compensatory resources. This is partially supported by the finding that that action selection led to a greater increase in response times for intermediate patients compared to the mild-motor predominant patients. The relatively small sample size of diffuse-malignant patients may explain why we did not find the same effect in this group. The lack of a differential effect of action selection demand on task performance between Parkinson’s disease patients and healthy controls eliminates the potential confound of different behavioural strategies, thereby improving the interpretability of the between-group effects on brain activity that we observed.^100^ This claim is further substantiated by a lack of between-group effects on response variability and switching.

This study was primarily set up to test for compensation through group and interindividual differences in activity amplitude. However, compensation may also manifest in other forms, such as altered spatial extent of activation.^101^ Further research is required to test the involvement of additional aspects of compensation.

The lack medication effects on parieto-premotor function suggest that cortical compensation in Parkinson’s disease does not depend on dopaminergic state and may therefore require support from alternative therapeutic approaches.^6^ However, despite clear improvements in motor symptoms following dopaminergic medication, we did not observe normalization of response times^102^ or basal ganglia function.^16^ One possible explanation is that patients were not completely OFF, due to long-lasting medication effects which are now well recognized.^103^ The effect of levodopa on motor symptoms is mediated by a short-duration response that alleviates symptoms within hours and a long-duration response that provides symptomatic relief over days to weeks.^104^ The long-duration response is particularly strong in early-stage Parkinson’s disease, meaning that the clinically defined OFF-state in which participants were assessed, defined as the withdrawal of dopaminergic medication for at least 12 h, may not have allowed the effect of medication to subside enough to detect subtle alterations in task performance and brain activity.^103^

Parkinson’s disease is associated with wide-spread brain atrophy that may have driven interindividual differences in compensatory capacity in this study.^87,105,106^ Contrary to these findings, we observed no grey matter volume alterations in parieto-premotor areas where differences in selection-related activity between clinical subtypes of Parkinson’s disease and healthy controls were located (Supplementary material). Instead, differences in grey matter volume were confined to a more inferiorly located network consisting of occipital, inferior parietal and temporal areas of the cortex, which is consistent with previous findings of brain atrophy in early-to-moderate stages of Parkinson’s disease (Supplementary Fig. 7).^106^ We therefore conclude that compensatory alterations in parieto-premotor cortex primarily reflect functional neuroplasticity rather than alterations in underlying brain structure.

## Conclusion

This study suggests that interindividual variability in Parkinson’s disease with respect to clinical subtype and symptom severity [bradykinesia and cognitive (dys)function] are determined, in part, by the degree to which parieto-premotor cortex is able to compensate for progressive basal ganglia dysfunction. Interventions and treatments that aim to modify the progression of Parkinson’s disease may benefit from focusing on enhancing the efficiency and maintenance of compensatory cortical processes in addition to restoring basal ganglia dysfunction.

## Supplementary Material

awad325_Supplementary_Data

## Data Availability

The data that support the findings of this study are available upon request only, to ensure the privacy of the participants. A data acquisition request can be sent to the corresponding author. All analysis code used in the present study is freely available at https://github.com/mejoh/Personalized-Parkinson-Project-Motor.
