# Supplementary material for: Clinical severity in Parkinson’s disease is determined by decline in cortical compensation
Source: Brain. 2023 Sep 27;147(3):871–86. doi: 10.1093/brain/awad325 (PMC10907095; doi:10.1093/brain/awad325)
Supplement: awad325_Supplementary_Data [file awad325_supplementary_data.pdf]

Supplementary material 1

Inclusion and exclusion criteria

Patients were eligible for participation if they had received a diagnosis of idiopathic Parkinson's disease from a certified neurologist, had 0-5 years disease duration, and were ≥ 18 years of age. It should be noted that the Personalized Parkinson Project cohort did not constitute a convenience sample, but rather aimed to include a cohort that represented real-life patients. Strict stratification criteria were applied to ensure a balanced inclusion of men and women, different age ranges (21-45; 46-55; 56-65; ≥66 years), and different disease durations (<2.5 years; ≥2.5 years). Healthy controls were eligible for participation if they were at least 40 years of age and were willing and able to return for a two-year follow-up measurement. All participants were able to read and understand Dutch, could comply with all aspects of the study protocol, and could provide written informed consent. Exclusion criteria included co-morbidities that would negatively influence the interpretability of parkinsonian disability, contraindications to MRI, pregnancy or breastfeeding, and nickel allergy (owing to the wearing of a study-related device). Additional exclusion criteria for healthy controls included co-morbidities that would negatively influence the interpretability of results from a comparison with PD patients. Further details can be found in the primary study protocol of the Personalized Parkinson Project.[1]

Measurements used for subtype classification

Motor symptoms were assessed with a composite score based on MDS-UPDRS II and III.[2] Cognitive function was assessed with a composite score derived from age-, education-, and sex-adjusted z-scores on the Benton Judgement of Line Orientation (visuospatial perception),[3] Brixton Spatial Anticipation Test (executive function),[4] Semantic Fluency Test (semantic fluency; 1 minute animal naming),[5] Symbol Digit Modalities Test (processing speed; 90 seconds, oral version),[6] Letter-Number Sequencing from the Wechsler Adult Intelligence Test – Fourth Edition (working memory),[7] and an average across subscores of the Rey Auditory Verbal Learning Test (episodic memory).[5,8] Z-scores from these six tests were averaged to compute a composite score of cognitive performance. REM sleep behavior disorder was assessed with the REM Sleep

Behavior Disorder Screening Questionnaire.[9] Autonomic function was assessed with the Scales for Outcomes in Parkinson's disease.[10]

Detailed description of the action selection task

Each trial began with the presentation of a fixation cross. After a random inter-stimulus interval of 2-4 seconds, the cross was replaced by a cue consisting of four circles, with each circle corresponding to a button on a response device. The circles were either filled or empty to indicate which responses were correct or incorrect, respectively. Participants were instructed to respond to cues by pressing a single button corresponding to a single filled circle. If multiple circles were highlighted, then participants were instructed to choose which circle to respond to. Only one response was allowed per trial. Each cue included either one, two, or three filled circles, thereby varying the number of response choices that participants were presented with on a trial-by-trial basis. Participants were encouraged to make use of all four buttons rather than selectively responding with only a subset of buttons, to vary the finger that was used to respond during trials where multiple response options were presented, and to respond as quickly and as accurately as possible. Cues remained on the screen for a maximum of 2 seconds or until a response was recorded and were immediately followed by the presentation of a new fixation cross. The task consisted of 132 trials and lasted for approximately 10 minutes, depending on performance. There were 60 one-choice trials (15 per finger), 30 two-choice trials, and 30 three-choice trials. The additional 12 trials consisted of 6 one-choice trials, 3 two-choice trials, and 3 three-choice trials where circles were outlined in red. Participants were instructed to withhold a response during these "catch" trials. Trials were presented in three blocks, each consisting of 44 trials. Out of these 44 trials, 20 were one-choice, 10 were two-choice, 10 were three-choice, and 4 were catch. The ordering of trial conditions within each block was pseudo-randomized. Blocks were separated by 20 seconds of rest. Prior to entering the scanner, participants practiced by performing one continuous block of 68 trials (30 one-choice trials, 15 two-choice trials, 15-three choice trials, 8 catch trials). Trials of one-, two-, and three-choice conditions were classified as correct, incorrect, or miss. Correct responses were defined as button presses executed within two seconds following cue onset that corresponded to a single, highlighted circle (in the case of catch trials, correct responses were defined as no response). Incorrect responses were defined as button presses executed within two seconds following cue onset that did not correspond to a highlighted circle.

Misses were defined as cues where no button press was executed within two seconds following cue onset. Patients were asked to perform the task with their most-affected hand. The responding hands of healthy controls were matched to the 56 patients who were assessed in an off-medicated state.

Preprocessing details

Registrations of functional images to anatomical space were estimated using linear transformations with boundary-based registration and six degrees-of-freedom.[11] Non-linear transformations were estimated from anatomical to MNI152Lin6Asym-space.[12,13] A single interpolation step was used to carry out all transformations in combination with motion correction[14] and slice time correction.[15] Confound time series were generated for framewise displacement and DVARS,[16] along with 24 motion derivatives. An anatomical principal component analysis was performed to derive time series for cerebrospinal fluid and white matter signal.[17] ICA-AROMA was used to derive time series of motion-related noise[18,19] whose classification was further refined using custom methodology (see section below). A series of discrete cosine-basis functions were derived for high-pass filtering (>0.008 Hz). Tremor regressors were generated from the accelerometry data of 69 patients.

Refinement of ICA-AROMA component selection

Additional steps were taken to ensure that the removal of confounding motion-related variability through ICA-AROMA during the first-level analysis did not adversely affect the estimation of task-related regressors. For each participant, the classification of ICA-AROMA components was refined in a multiple regression analysis where each noise time series was modelled as a function of task regressors for choice and catch conditions. Time series that shared more than 5% explained variance, as assessed by the r^2 of the regression model, were reclassified as non-noise and subsequently left out of the first-level design.

Quantification of tremor

For all patients, tremor severity was quantified using a three-axial accelerometer placed on the dorsum of the most-affected hand. Accelerometry preprocessing involved detrending, demeaning,

transformation to scan-to-scan tremor power at peak frequency, and log-transformation, after which the tremor signal was convolved with a canonical hemodynamic response function.[20] The resulting tremor regressors were added to the first-level models of 69 patients whose tremor was confirmed through visual inspection.

Exclusions and final sample sizes for analyses of behavioral performance and task-related activity

All healthy controls had full data. For patients, final sample sizes were determined separately for analyses of task performance and brain activity. Exclusions were carried out sequentially in the order that they are reported. Note that most analyses depended on data availability along multiple data streams and were therefore subjected to further constraints with respect to final sample size.

Behavioral performance

Out of 367 patients assessed in the on-medicated state, 14 were excluded due to misdiagnosis, 5 had missing behavioral data, and 11 were excluded due to poor performance. This led to a total sample size of 337 patients. 50 of these patients had data from assessments in the off-medicated state, 306 had sufficient data to carry out subtype classification, 335 had usable bradykinesia scores, and 321 had usable cognitive composite scores.

Task-related activity

Out of 367 patients assessed in the on-medicated state, 14 were excluded due to misdiagnosis, 7 had missing imaging data, 9 had insufficient behavioral data to carry out 1st-level analyses, 3 were lost due to technical issues, 8 were excluded based on excessive movement, and 3 were excluded due to poor accuracy. This led to a total sample size of 323. 48 of these patients had data from assessments in the off-medicated state, 296 had sufficient data to carry out subtype classification, 322 had usable bradykinesia scores, 307 had usable cognitive composite scores.

Supplementary material 2

Task-related activity across groups

Task-related activity was investigated across groups to generate activation maps of motor-activity, catch-related activity, intermediate selection-related activity, and high action selection-related activity.

Motor-related activity

A conjunction analysis of one-choice, two-choice, and three-choice activity yielded a network of cerebellar, visual, parietal, insular, and sensorimotor activity (Supp. Fig. 1A). Sensorimotor activity was more extensive in the left hemisphere, as would be expected given that this was the responding side of all participants, either naturally or as a result of horizontal flipping of contrast images.

Catch-related activity

Catch-related activity primarily captured visual, parietal, insular, and prefrontal activity (Supp. Fig. 1B).

Action selection-related activity

Moderate (Supp. Fig. 1C) and high (Supp. Fig. 1D) demand on action selection elicited increased activity in a frontoparietal network and decreased activity in regions involved in the default mode network. This is consistent with the idea that action selection led to the recruitment of additional cognitive processing beyond simple motor responses.

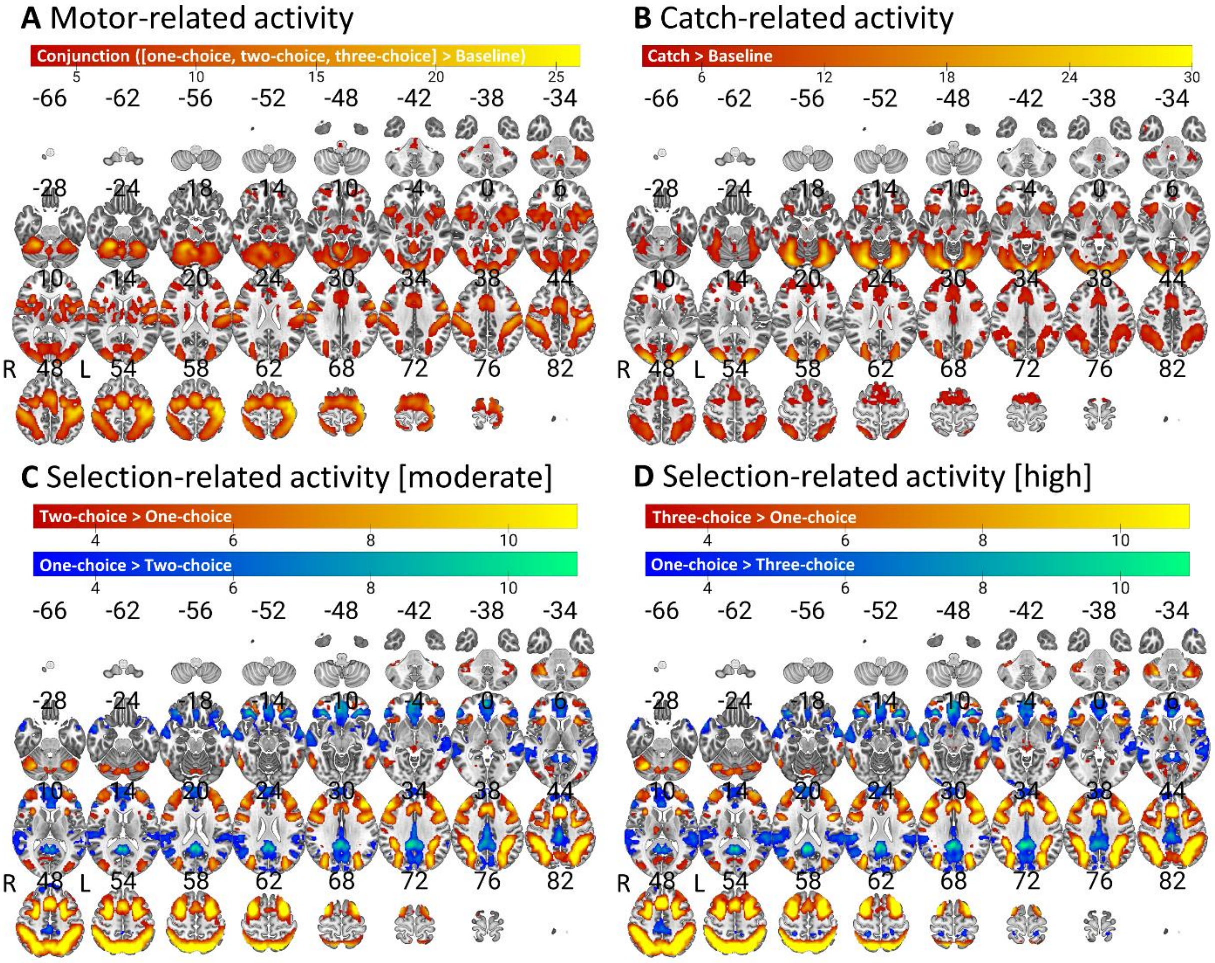

Supplementary figure 1 Task-related activity across patients and healthy controls. (A) Sensorimotor network activation common to all levels of action selection demand. (B) Response withholding preferentially activates visual and prefrontal cortex. (C) Moderate and (D) high action selection demand leads to activation of the frontoparietal network and deactivation of the default mode network.

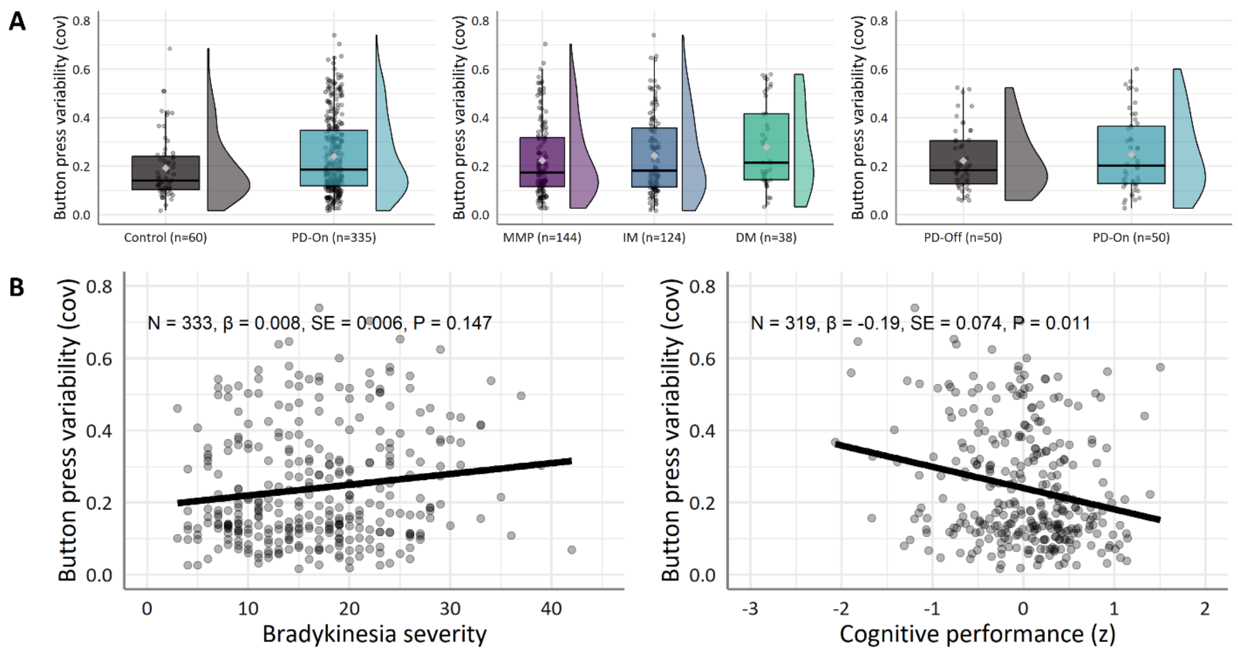

Supplementary figure 2 Effects on response variability. (A) Group comparisons. (**B)** Clinical correlations. Cov = Coefficient of variation.

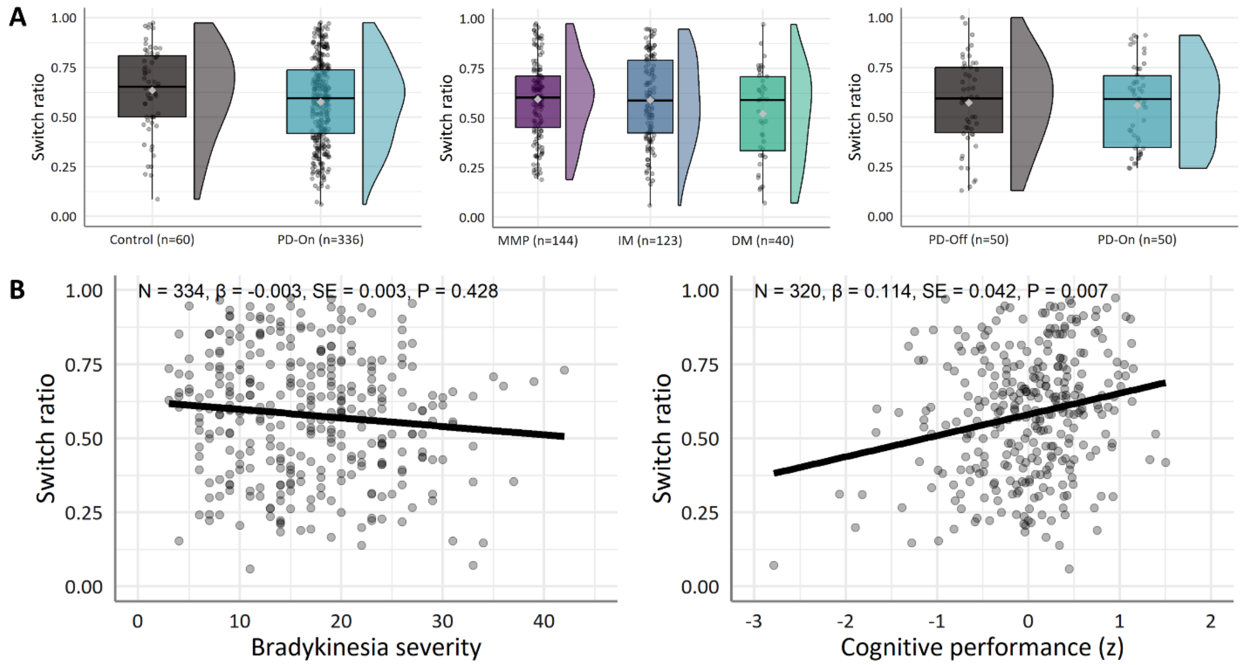

Supplementary figure 3 Effects on switching ratios. **(A)** Group comparisons. **(B)** Clinical correlations.

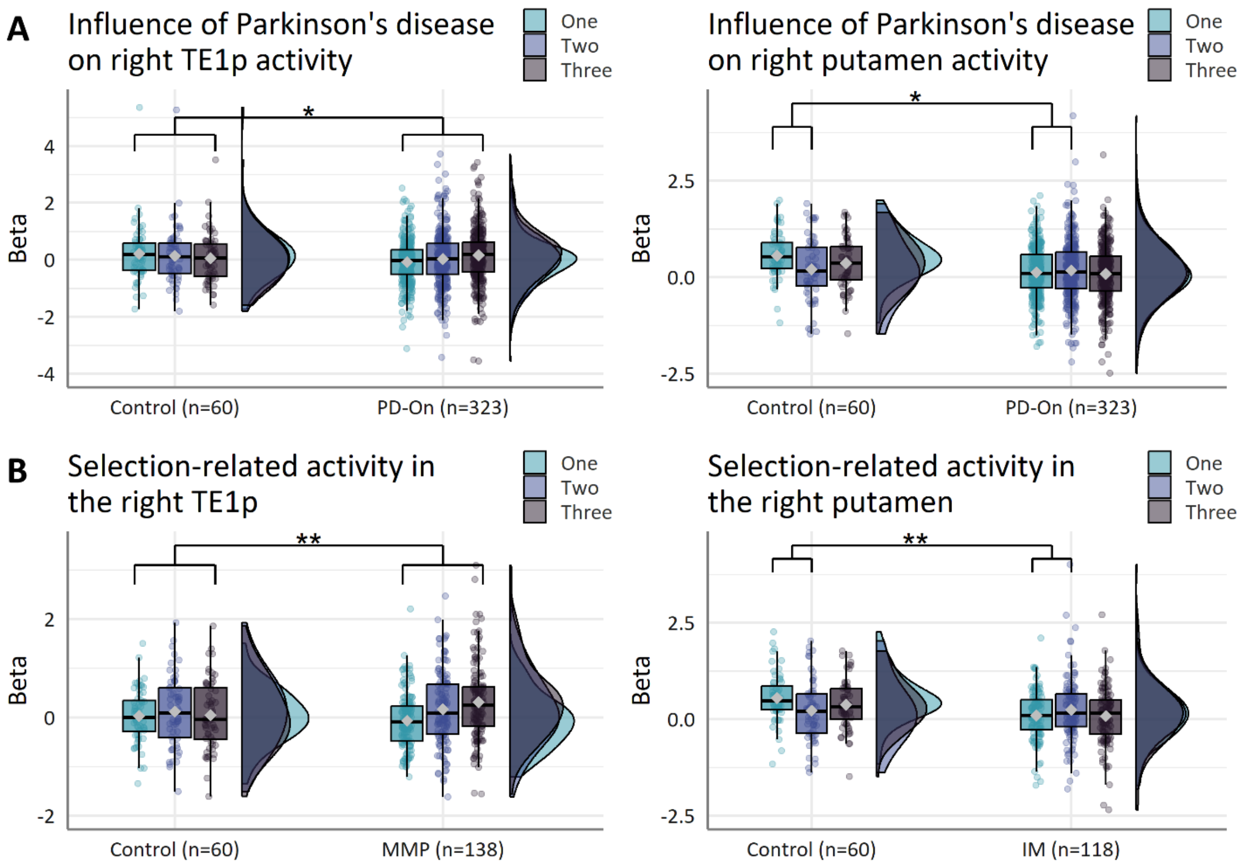

Supplementary figure 4 Differences in selection-related activity between patients and controls. (A) Patients show increased selection-related activation in the right middle temporal gyrus (left) and reduced selection-related activity in the right putamen (right) compared to healthy controls. **(B)** Alterations in right middle temporal gyrus (left) and in right putamen (right) was specific to the mild-motor predominant and intermediate subtypes, respectively.

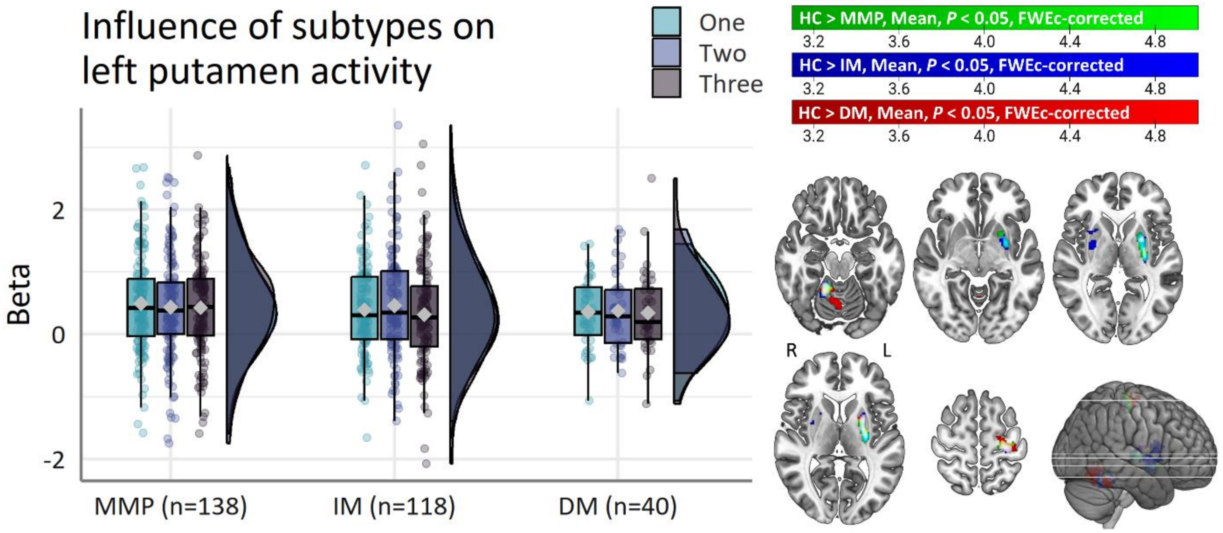

Supplementary figure 5 Subtypes show similar deficits in basal ganglia function. Subtypes show no differences in putamen activity in the most affected hemisphere (left). Compared to controls, all subtypes show decreased motor-related activity in a common network comprising the putamen, primary somatosensory cortex, and cerebellum (right).

Supplementary material 3

Separate brain – clinical correlations in motor and cognitive domains

Our correlation analysis of the relationship between clinical severity and motor- and selection-related brain activity focused on quantifying the independent contributions of bradykinesia and cognitive performance. Here, we performed a post hoc analysis quantifying correlations between brain activity and each clinical measure, separately.

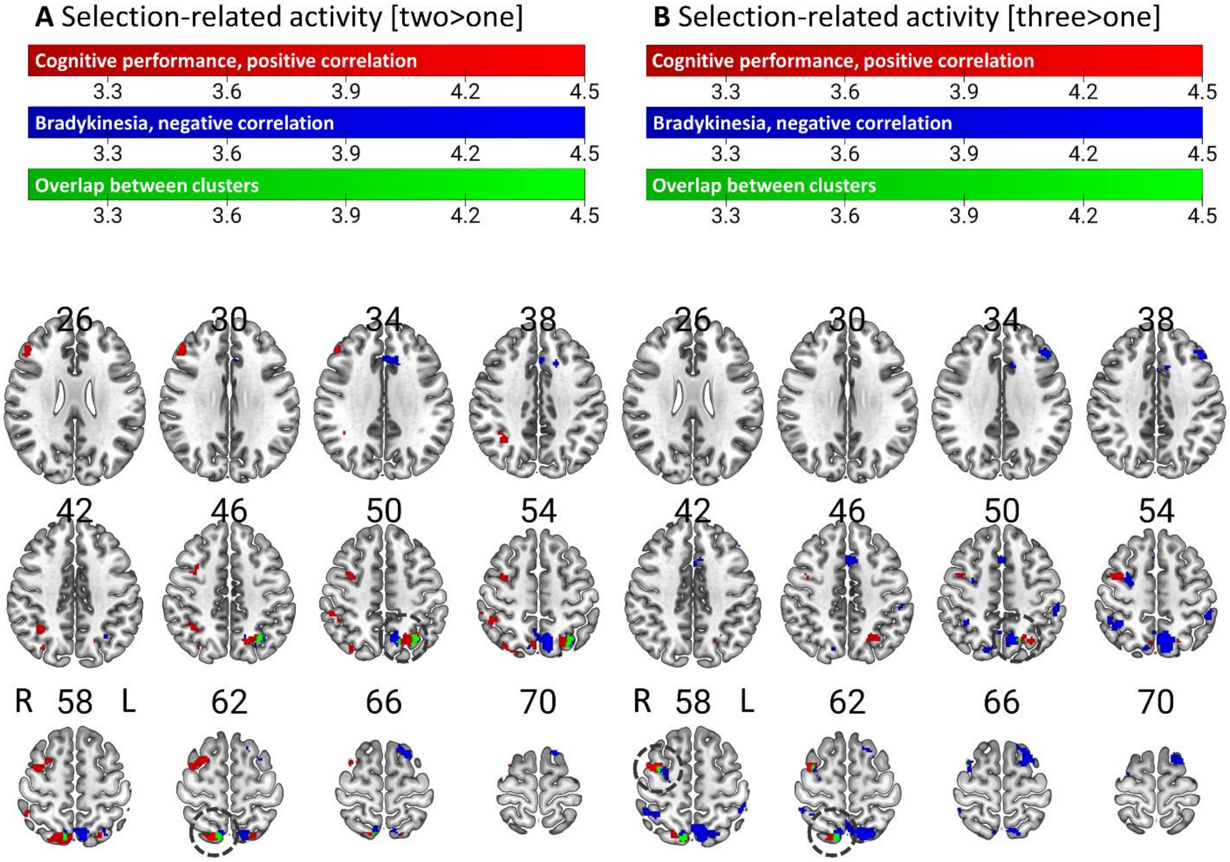

Supplementary figure 6. Domain-specific correlations between clinical severity and selection-related activity. (A) Correlations with moderate selection-related activity reveals minor overlap in left and right superior parietal lobule. **(B)** Correlations with high selection-related activity reveals minor overlap in the right superior parietal lobule and premotor cortex.

Bradykinesia severity

Bradykinesia severity was associated with selection-related activity in a network of parieto-premotor regions, primarily in the left hemisphere (Supp. Fig. 7). It may be argued that increased parieto-premotor activation could reflect alterations in saccadic eye-movement control rather than the activation of compensatory processes.[21,22] Arguing against this interpretation for correlations related to bradykinesia, we found no clusters that centred on typical saccade control regions, such as the frontal and posterior eye fields.

Supplementary table 1 Voxel-wise correlations of bradykinesia severity

Anatomical label (% cluster volume)	Area	*P*-value (FWEc-corrected)	Cluster extent (voxels)	Max *T*-value	MNI: X, Y, Z
**Mean > Baseline**					
**Bradykinesia, negative correlation**					
L precentral gyrus (43%)	6a	0.008	129	4.26	-25,-11,54
**Two > One**					
**Bradykinesia, negative correlation**					
L superior parietal lobule (69%)	7A	<0.001	573	5.15	-9,-61,54
L superior frontal gyrus (73%)	6ma	0.001	179	4.18	-23,7,68
R superior parietal lobule (46%)	7PC	0.001	171	4.62	35,-47,54
R superior parietal lobule (89%)	7P	0.001	160	4.77	11,-67,60
R superior frontal gyrus (57%)	6a	0.002	155	4.19	29,-5,56
L supplementary motor area (57%)	p32pr	0.004	136	4.01	-3,15,46
L inferior parietal lobule (84%)	IP2	0.012	110	3.90	-52,-39,56
L middle frontal gyrus (100%)	46	0.035	88	4.84	-46,29,38
**Three > One**					
**Bradykinesia, negative correlation**					
L superior parietal lobule (79%)	7A	<0.001	326	4.65	-9,-36,54
L superior parietal lobule (83%)	MIP	0.006	125	4.13	-31,-63,48
L middle cingulate cortex (59%)	a32pr	0.018	100	4.19	-17,19,38
R superior parietal lobule (99%)	7Am	0.032	89	4.45	9,-67,62
L superior frontal gyrus (66%)	6ma	0.032	89	4.24	-15,15,68

Anatomical labels were derived from the Anatomy Toolbox v3.0. Area labels were derived from the Glasser atlas.

Cognitive performance

Like bradykinesia severity, correlations with cognitive performance are located primarily in a network of parieto-premotor regions (Supp. Fig. 7). However, these regions tended to be adjacent to the regions implicated in bradykinesia rather than overlapping with them, and were

preferentially located in the right hemisphere. Here, we observed a single cluster in the frontal eye fields of the right hemisphere. While we cannot exclude the possibility this effect may relate to saccadic eye-movement control, we note that the frontal eye fields also play an important role in goal-directed guidance of attention, an important aspect of general cognitive function.[23,24]

Supplementary table 2 Voxel-wise correlations of cognitive performance

Anatomical label (% cluster volume)	Area	*P*-value (FWEc-corrected)	Cluster extent (voxels)	Max *T*-value	MNI: X, Y, Z
**Mean > Baseline**					
**Cognitive performance, positive correlation**					
R inferior parietal lobule (43%)	7PC	<0.001	248	4.59	39,-39,54
L postcentral gyrus (82%)	M1	0.004	160	4.03	-33,-29,48
L middle cingulate cortex (55%)	p32pr	0.032	106	4.11	-3,17,42
L precentral gyrus (100%)	6a	0.037	102	4.44	-31,-13,50
**Two > One**					
**Cognitive performance, positive correlation**					
R middle frontal gyrus (83%)	FEF	0.001	170	4.77	35,-1,58
L superior parietal lobule (97%)	MIP	0.015	105	3.81	-29,-63,48
R superior parietal lobule (60%)	7P	0.015	105	3.80	11,-71,60
**Three > One**					
**Cognitive performance, positive correlation**					
L superior parietal lobule (91%)	MIP	<0.001	272	4.12	-17,-65,46
R superior parietal lobule (51%)	7P	<0.001	257	4.93	13,-69,64
R middle frontal gyrus (50%)	i6-8	<0.001	227	4.16	25,7,62
R inferior parietal lobule (41%)	IP2	0.010	120	4.10	33,-53,44
R middle frontal gyrus (59%)	9-46v	0.022	103	4.28	47,36,30
R inferior parietal lobule (87%)	PFm	0.044	88	3.80	45,-45,54
**Cognitive performance, negative correlation**					
L cuneus	V2	0.027	98	4.65	-9,-94,20

Anatomical labels were derived from the Anatomy Toolbox v3.0. Area labels were derived from the Glasser atlas.

Overlap between domains

Interestingly, there was little overlap between cluster related to bradykinesia severity and cognitive performance.

Supplementary material 4

Do individual differences in cortical compensatory capacity depend on underlying differences in reserve?

Cerebral compensation refers to functional adaptations that enable patients to meet behavioral demands despite the presence of some form of underlying dysfunction in the mechanisms that ordinarily support behavioral performance, particularly when the task at hand is relatively demanding. Compensation can be distinguished from the related concept of reserve, which has been conceptualized as a pre-morbid accumulation of neural resources that partially offsets symptomatic decline resulting from progressive pathology.[25–29] Patients with higher reserve may experience less severe symptoms compared to patients with low reserve despite having similar levels of pathology because their brains have more resources to draw from. Reserve typically manifests as a trait-like property in the sense that its effect on behavior tends to be present across multiple clinical domains. In contrast to compensation, which can be observed as a recruitment of additional resources in response to increasing task demands (through upregulation, selection or recruitment of additional mechanisms), reserve can only be estimated indirectly through proxy measures that are argued to have conferred beneficial effects on neural resources prior to disease onset. For example, higher levels of educational attainment may strengthen brain structure and function, thereby increasing resistance to pathology. We should note that some researchers define reserve in terms of both behavior (cognitive[28] or motor[26] reserve) and underlying brain function (neural reserve).[28] For example, it has been suggested that cognitive reserve denotes factors that reduce susceptibility to cognitive decline, whereas neural reserve denotes factors that reduce susceptibility to decline in brain function. According to this conceptualization, cognitive reserve can be supported by both neural reserve and cerebral compensation. However, the distinction between behavioral reserve and neural reserve can appear somewhat artificial, given that behavior depends on the brain. For the purposes of this study, we focus solely on compensation and reserve in the context of brain activity, where the two concepts can be more clearly distinguished from each other.

Cerebral compensation can be disentangled from reserve by assessing the relationship between brain activity and clinical severity while simultaneously controlling for reserve proxies.

Individual differences in cerebral compensation should be more strongly related to behaviour than with reserve proxies and can be expected to be sensitive to manipulations of task difficulty. Neural reserve should be more related to proxy measures of reserve, and, given its trait-like nature, may not be as sensitive to manipulations of task difficulty. However, compensation may depend on reserve. It is conceivable that higher levels of reserve may enable patients to more effectively recruit compensatory resources. When investigating cerebral compensation, it is therefore useful to explore whether individual differences in compensatory activity depend on underlying reserve. In our between-subtype comparisons and brain-clinical association analyses we already controlled for several proxies of reserve, such as age,[30] sex,[31,32] and years of education,[33] which are considered prominent contributors to reserve in relation to both motor and cognitive domains.[26,34] However, there are additional proxy measures of reserve that may probe different aspects of reserve in relation to Parkinson's disease. For example, there is evidence suggesting lower body-mass index, more engagement in physical activities, lower non-motor burden, and smoking history may decrease susceptibility to the behavioral effects of pathological decline.[26,35,36]

We therefore performed an exploratory re-analysis of our between-subtype comparisons and brain-clinical association analyses to test whether the relationship between increased task-related activity and clinical severity (as quantified through subtyping and clinical scores of symptom severity) remained after controlling for additional reserve proxies: body-mass index (continuous measure), smoking history (no/yes), engagement in physical activity (Physical Activity Scale for the Elderly [PASE];[37] continuous measure), and non-motor burden (MDS-UPDRS part I;[2] continuous measure). Body-mass index and physical activity should be viewed as approximations of pre-morbid lifestyle choices given that they were measured at baseline.

In comparisons between subtypes, we observed that regions showing stronger selection-related activity in both mild-motor predominant and intermediate subtypes compared to the diffuse-malignant subtype remained significant (Supp. Table 1). Furthermore, we observed additional parietal clusters of stronger motor-related activity in the mild-motor predominant subtype compared to the diffuse-malignant subtype. This supports our hypothesis that upregulation of parieto-premotor activity is more strongly related to compensation than reserve.

The inverse relationship between selection-related activity and bradykinesia severity remained significant in parieto-premotor cortex, but only at moderate action selection demand

(Supp. Table 1). Correlations between selection-related activity and cognitive performance did not remain significant, with the exception of a cluster in the secondary visual cortex. However, better cognitive performance was now associated with greater motor-related activity in primary somatosensory cortex and supplementary motor area. In combination, these findings suggest that reserve may influence the degree to which compensatory cortical adaptations contribute to counteracting specific clinical domains.

Supplementary table 3 Voxel-wise analyses accounting for additional proxy measures of reserve

Anatomical label (% cluster volume)	Area	*P*-value (FWEc-corrected)	Cluster extent (voxels)	Max *T*-value	MNI: X, Y, Z
**Influence of subtype**					
**Mild-motor predominant > Intermediate, Mean > Baseline**					
R postcentral gyrus	2	0.001	213	3.92	43,-25,46
**Mild-motor predominant > Diffuse-malignant, Mean > Baseline**					
R postcentral gyrus	2	<0.001	459	5.12	45,-23,42
R superior parietal lobule	7A	0.003	168	4.29	25,-51,72
L inferior parietal lobule	IP2	0.003	164	4.60	-54,-37,52
**Mild-motor predominant > Diffuse-malignant, Three > One**					
R middle frontal gyrus (74%)	i6-8	<0.001	400	4.60	33,3,52
R inferior parietal lobule (82%)	PFm	0.005	153	4.17	47,-55,38
R superior parietal lobule (96%)	7P	0.005	149	4.31	5,-59,52
**Intermediate > Diffuse-malignant, Three > One**					
L middle frontal gyrus (68%)	6a	0.05	94	4.58	-21,3,56
**Brain – clinical associations**					
**Mean > Baseline**					
**Cognitive performance, positive**					
R postcentral gyrus (72%)	1	<0.001	311	4.15	39,-39,56
L postcentral gyrus (72%)	M1	0.001	205	4.25	-33,-29,48
L supplementary motor area (84%)	SCEF	0.016	117	4.20	1,-3,64
**Two > One**					
**Bradykinesia, negative**					
L superior parietal lobule (92%)	7Am	<0.001	216	4.07	-9,-61,52
L inferior parietal lobule (74%)	1	0.003	141	3.94	-52,-39,56
L superior frontal gyrus (91%)	6ma	0.012	109	4.07	-21,5,68
**Three > One**					
**Cognitive performance, negative**					
L cuneus (61%)	V2	0.007	120	4.43	-9,-94,20

Anatomical labels were derived from the Anatomy Toolbox v3.0. Area labels were derived from the Glasser atlas.

Supplementary material 5

Voxel-based morphometry

Processing and statistical analysis of gray matter volume in regions showing group effects on brain activity

Functional alterations in Parkinson's disease may be associated with underlying patterns of structural atrophy. Voxel-based morphometry (VBM)[38] was used to estimate gray matter volume in brain regions that showed significant differences in brain activity between patients and controls and between subtypes. Significant results from subtype comparisons were followed with post hoc comparisons between subtypes and controls. The Computational Anatomy Toolbox[39] (CAT; version 12.8) was used to segment T1-weighted structural images into gray matter, white matter, and cerebrospinal fluid. Gray matter segmentations were normalized to a study-specific gray matter template that was generated with geodesic shooting nonlinear image registration[40] based on 59 healthy controls and 59 randomly selected PD patients. The normalized gray matter images were subsequently modulated by Jacobian determinants to account for the non-linear component of the normalization procedure.

Average gray matter volumes were separately extracted from networks consisting of clusters that showed a significant effects of GROUP and GROUP × CHOICE on brain activity. Volumes were extracted from contralateral networks for participants whose first-level contrast had been flipped to ensure correspondence between functional and structural analyses. The grand-average gray matter volume of each network was calculated per participant, yielding a single metric of gray matter volume per network. One-way ANCOVAs were used to test the effect of GROUP on gray matter volume within each network. Total intracranial volume, age, years of education, and dominant hand were modelled as covariates of non-interest. Comparisons that yielded significant between-group differences were followed with an exploratory analysis of the association between gray matter volume and clinical severity (bradykinesia and cognitive performance) in all patients with Parkinson's disease. Whole-brain voxel-wise comparisons were conducted to explore more extensive differences in gray matter volumes.

Group comparisons of gray matter volume

Patients versus controls

The region of the middle temporal cortex where patients showed increased selection-related activation compared to controls (patient>control, three-choice>one-choice) also showed decreased gray matter volume (control>patient [$F(1)=8.5$, $P=0.004$, $\eta^2_p=0.02$]). Lower gray matter volume in this region was associated with worse cognitive performance ($\beta=-0.027$, SE=0.009, $F(1)=8.8$, $P=0.012$).

Voxel-wise whole-brain comparisons revealed decreased gray matter volume in patients compared to controls in a network consisting of large areas of visual, temporal, orbitofrontal, posterior parietal cortex, and the anterior striatum (Supp. Fig. 7A). Parietal cortex and striatal atrophy was primarily lateralized to the left hemisphere.

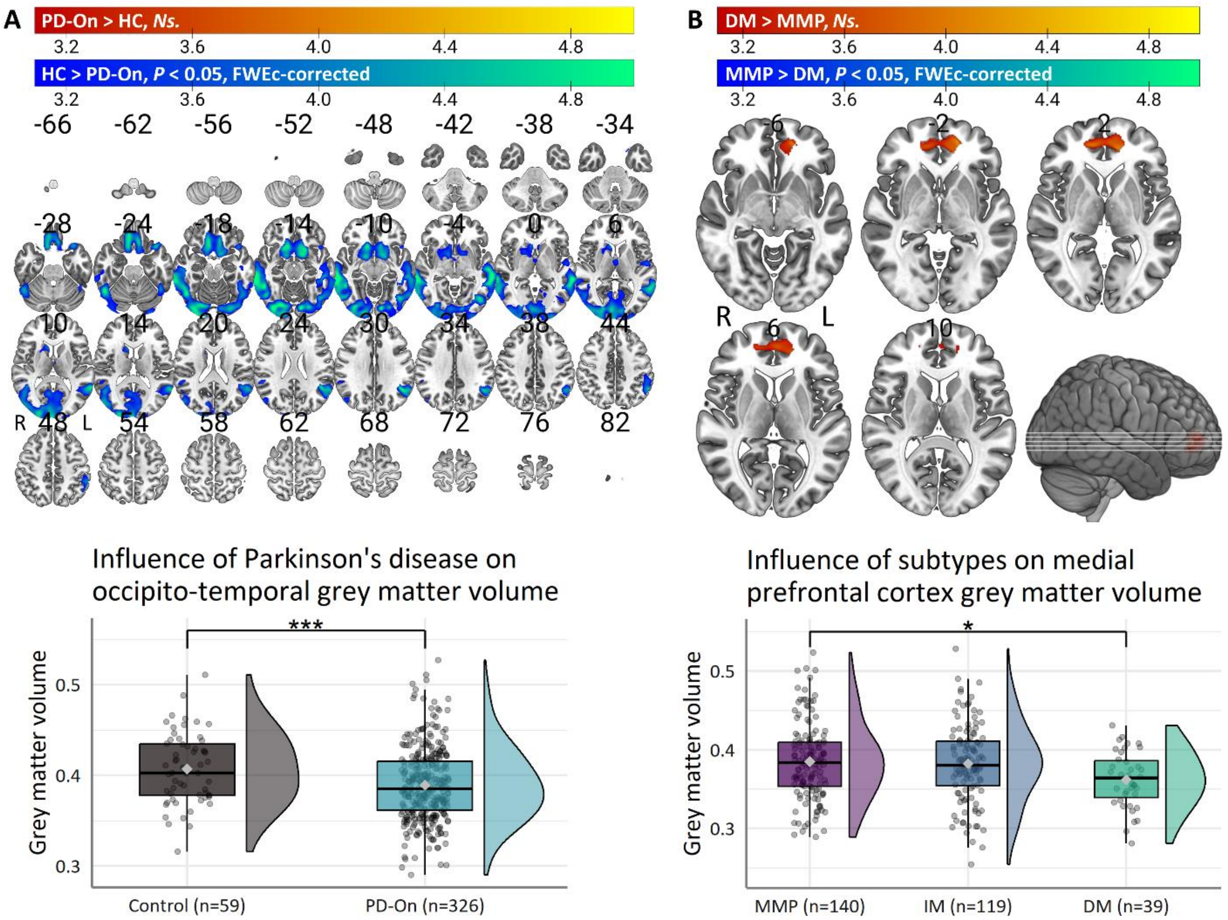

Supplementary figure 7 Gray matter volume reductions in Parkinson's disease. (A) Voxel-wise comparison of gray matter volume between patients and healthy controls reveals a network of atrophy consisting of large regions of visual, temporal, inferior parietal, and orbitofrontal cortex, as well as anterior regions of the striatum. **(B)** Mild-motor predominant patients show larger gray matter volumes in medial prefrontal cortex compared to diffuse-malignant patients.

Subtypes

The region of the postcentral gyrus (area S1) where the diffuse-malignant subtype showed decreased motor-related activity compared to the mild-motor predominant subtype (mild-motor predominant>diffuse-malignant, mean>baseline) also showed a decrease in gray matter volume (mild-motor predominant>diffuse-malignant [$F(1)=5.6$, $P=0.019$, $\eta^2_p=0.04$]). There were no correlations with clinical severity.

The network where the mild-motor predominant subtype showed increased selection-related activity compared to controls (mild-motor predominant>control, three-choice>one-choice) showed a decrease in gray matter volume (control>mild-motor predominant [$F(1)=8.2$, $P=0.005$, $\eta^2_p=0.05$]). Lower gray matter volume in this network was associated with worse cognitive performance ($\beta=0.017$, $SE=0.006$, $T(285)=2.6$, $P=0.008$) and at trend-level with higher bradykinesia severity ($\beta=-0.0010$, $SE=0.0005$, $T(285)=1.9$, $P=0.066$). A post hoc analysis of each separate region of the network showed that this correlation was driven primarily by the region of middle frontal gyrus (control>mild-motor predominant [$F(1)=8.1$, $P=0.005$, $\eta^2_p=0.05$]) where patients showed an increase in activity compared to controls.

Voxel-wise whole-brain comparisons revealed decreased gray matter volume in left anterior cingulate gyrus (p32) in the mild-motor predominant subtype compared to the diffuse-malignant subtype (Supp. Fig. 7B; mild-motor predominant>diffuse-malignant [*P*, *FWEc-corrected*=?, cluster extent=?, max *t*=?, XYZ=]). No other whole-brain comparisons yielded significant group differences.

Supplementary material 6

The influence of medication

Behavioral performance

Response times increased as a function of action selection demand (Supp. Fig. 4; main effect of CHOICE [$\chi^2(2)$=22.9, P<0.001, η^2_p=0.07]; moderate>low [*log-ratio*=1.06, *SE*=0.009, *t-ratio*(843)=7.0, *P*<0.001], high>low [*log-ratio*=1.06, *SE*=0.009, *t-ratio*(843)=6.4, *P*<0.001]). There was no effect of medication on response times (*P*=0.36).

Error rates increased as a function of action selection demand (Supp. Fig. 4; main effect of CHOICE [$\chi^2(2)$=9.5, p=0.009]; low>high [*OR*=2.5, *SE*=0.53, *Z-ratio*=4.4, *P*<0.001], moderate>high [*OR*=2.2, *SE*=0.52, *Z-ratio*=3.4, *P*=0.002]). There was no effect of medication on error rates (*P*=0.56).

There was no effect of medication on response perseverance (Supp. Fig. 2G; *P*=0.72) or switching (Supp. Fig. 2H; *P*=0.97).

Brain activity

There were no effects of medication on motor- or selection-related activity**.**

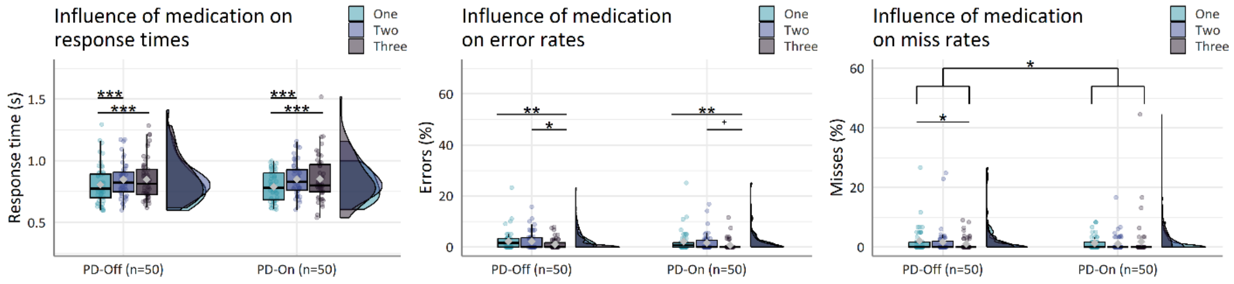

Supplementary figure 8 Influence of medication on response times (left), error rates (middle), and miss rates (right).

References

1. Bloem BR, Marks WJ, Silva de Lima AL, et al. The Personalized Parkinson Project: examining disease progression through broad biomarkers in early Parkinson's disease. *BMC Neurol*. 2019;19(1):1-10. doi:10.1186/s12883-019-1394-3

2. Goetz CG, Tilley BC, Shaftman SR, et al. Movement Disorder Society-Sponsored Revision of the Unified Parkinson's Disease Rating Scale (MDS-UPDRS): Scale presentation and clinimetric testing results. *Mov Disord*. 2008;23(15):2129-2170. doi:10.1002/mds.22340

3. Benton AL. Visuospatial Judgment. *Arch Neurol*. 1978;35(6):364. doi:10.1001/archneur.1978.00500300038006

4. Burgess PW, Shallice T. The Hayling and Brixton Tests. *Bury St Edmunds Thames Val Test Co*. Published online 1996.

5. Bouma A, Mulder J, Lindeboom J, Schmand B. *Handboek Neuropsychologische Diagnostiek*.; 2012.

6. Smith A. Symbol Digit Modalities Test. 1982. *West Psychol Serv Los Angeles*. Published online 1982.

7. Weschler D. Weschler Adult Intelligence Scale. Published online 1955.

8. Saan RJ, Deelman BG. De 15-Woordentests A en B. Een voorlopige handleiding (Intern rapport). *Groningen AZG, afd Neuropsychol*. Published online 1986.

9. Stiasny-Kolster K, Mayer G, Schäfer S, Möller JC, Heinzel-Gutenbrunner M, Oertel WH. The REM sleep behavior disorder screening questionnaire - A new diagnostic instrument. *Mov Disord*. 2007;22(16):2386-2393. doi:10.1002/mds.21740

10. Visser M, Marinus J, Stiggelbout AM, van Hilten JJ. Assessment of autonomic dysfunction in Parkinson's disease: The SCOPA-AUT. *Mov Disord*. 2004;19(11):1306-1312. doi:10.1002/mds.20153

11. Greve DN, Fischl B. Accurate and robust brain image alignment using boundary-based registration. *Neuroimage*. 2009;48(1):63-72. doi:10.1016/j.neuroimage.2009.06.060

12. Evans AC, Janke AL, Collins DL, Baillet S. Brain templates and atlases. *Neuroimage*. 2012;62(2):911-922. doi:10.1016/j.neuroimage.2012.01.024

13. Avants BB, Tustison NJ, Song G, Cook PA, Klein A, Gee JC. A reproducible evaluation of ANTs similarity metric performance in brain image registration. *Neuroimage*. 2011;54(3):2033-2044. doi:10.1016/j.neuroimage.2010.09.025

14. Jenkinson M, Bannister P, Brady M, Smith S. Improved Optimization for the Robust and Accurate Linear Registration and Motion Correction of Brain Images. *Neuroimage*. 2002;17(2):825-841. doi:10.1006/nimg.2002.1132

15. Cox RW, Hyde JS. Software tools for analysis and visualization of fMRI data. *NMR Biomed*. 1997;10(4-5):171-178. doi:10.1002/(SICI)1099-1492(199706/08)10:4/5<171::AID-NBM453>3.0.CO;2-L

16. Power JD, Mitra A, Laumann TO, Snyder AZ, Schlaggar BL, Petersen SE. Methods to detect, characterize, and remove motion artifact in resting state fMRI. *Neuroimage*. 2014;84:320-341. doi:10.1016/j.neuroimage.2013.08.048

17. Behzadi Y, Restom K, Liau J, Liu TT. A component based noise correction method (CompCor) for BOLD and perfusion based fMRI. *Neuroimage*. 2007;37(1):90-101. doi:10.1016/j.neuroimage.2007.04.042

18. Pruim RHR, Mennes M, van Rooij D, Llera A, Buitelaar JK, Beckmann CF. ICA-AROMA: A robust ICA-based strategy for removing motion artifacts from fMRI data. *Neuroimage*. 2015;112:267-277. doi:10.1016/j.neuroimage.2015.02.064

19. Pruim RHR, Mennes M, Buitelaar JK, Beckmann CF. Evaluation of ICA-AROMA and alternative strategies for motion artifact removal in resting state fMRI. *Neuroimage*. 2015;112:278-287. doi:10.1016/j.neuroimage.2015.02.063

20. Helmich RC, Janssen MJR, Oyen WJG, Bloem BR, Toni I. Pallidal dysfunction drives a cerebellothalamic circuit into Parkinson tremor. *Ann Neurol*. 2011;69(2):269-281. doi:10.1002/ana.22361

21. Chan F, Armstrong IT, Pari G, Riopelle RJ, Munoz DP. Deficits in saccadic eye-movement control in Parkinson’s disease. *Neuropsychologia*. 2005;43(5):784-796.

doi:10.1016/j.neuropsychologia.2004.06.026

22. Ptak R, Müri RM. The parietal cortex and saccade planning: Lessons from human lesion studies. *Front Hum Neurosci*. 2013;7(JUN):1-11. doi:10.3389/fnhum.2013.00254

23. Duncan J. The multiple-demand (MD) system of the primate brain: mental programs for intelligent behaviour. *Trends Cogn Sci*. 2010;14(4):172-179. doi:10.1016/j.tics.2010.01.004

24. Ptak R. The frontoparietal attention network of the human brain: Action, saliency, and a priority map of the environment. *Neuroscientist*. 2012;18(5):502-515. doi:10.1177/1073858411409051

25. Cabeza R, Albert M, Belleville S, et al. Maintenance, reserve and compensation: the cognitive neuroscience of healthy ageing. *Nat Rev Neurosci*. 2018;19(11):701-710. doi:10.1038/s41583-018-0068-2

26. Chung SJ, Lee JJ, Lee PH, Sohn YH. Emerging concepts of motor reserve in Parkinson's disease. *J Mov Disord*. 2020;13(3):171-184. doi:10.14802/jmd.20029

27. Barulli D, Stern Y. Efficiency, capacity, compensation, maintenance, plasticity: Emerging concepts in cognitive reserve. *Trends Cogn Sci*. 2013;17(10):502-509. doi:10.1016/j.tics.2013.08.012

28. Stern Y. Cognitive reserve in ageing and Alzheimer's disease. *Lancet Neurol*. 2012;11(11):1006-1012. doi:10.1016/S1474-4422(12)70191-6

29. Stern Y, Barnes CA, Grady C, Jones RN, Raz N. Brain reserve, cognitive reserve, compensation, and maintenance: operationalization, validity, and mechanisms of cognitive resilience. *Neurobiol Aging*. 2019;83:124-129. doi:10.1016/j.neurobiolaging.2019.03.022

30. Kempster PA, O'Sullivan SS, Holton JL, Revesz T, Lees AJ. Relationships between age and late progression of Parkinson's disease: a clinico-pathological study. *Brain*. 2010;133(6):1755-1762. doi:10.1093/brain/awq059

31. Picillo M, Nicoletti A, Fetoni V, Garavaglia B, Barone P, Pellecchia MT. The relevance of gender in Parkinson's disease: a review. *J Neurol*. 2017;264(8):1583-1607.

doi:10.1007/s00415-016-8384-9

32. Iwaki H, Blauwendraat C, Leonard HL, et al. Differences in the Presentation and Progression of Parkinson's Disease by Sex. *Mov Disord*. 2021;36(1):106-117. doi:10.1002/mds.28312

33. Hindle J V., Martyr A, Clare L. Cognitive reserve in Parkinson's disease: A systematic review and meta-analysis. *Park Relat Disord*. 2014;20(1):1-7. doi:10.1016/j.parkreldis.2013.08.010

34. Stern Y. Cognitive reserve in ageing and Alzheimer's disease. *Lancet Neurol*. 2012;11(11):1006-1012. doi:10.1016/S1474-4422(12)70191-6

35. Noyce AJ, Bestwick JP, Silveira-Moriyama L, et al. Meta-analysis of early nonmotor features and risk factors for Parkinson disease. *Ann Neurol*. 2012;72(6):893-901. doi:10.1002/ana.23687

36. Bloem BR, Okun MS, Klein C. Parkinson's disease. *Lancet*. 2021;397(10291):2284-2303. doi:10.1016/S0140-6736(21)00218-X

37. Washburn RA, Smith KW, Jette AM, Janney CA. The physical activity scale for the elderly (PASE): Development and evaluation. *J Clin Epidemiol*. 1993;46(2):153-162. doi:10.1016/0895-4356(93)90053-4

38. Mechelli A, Price CJ, Friston KJ, Ashburner J. Voxel-based morphometry of the human brain: methods and applications. *Curr Med …*. 2005;1(2):105-113. doi:10.2174/1573405054038726

39. Gaser C, Dahnke R, Kurth K, Luders E, Alzheimer's Disease Neuroimaging Initiative. *A Computational Anatomy Toolbox for the Analysis of Structural MRI Data.*; 2022.

40. Ashburner J, Friston KJ. Diffeomorphic registration using geodesic shooting and Gauss–Newton optimisation. *Neuroimage*. 2011;55(3):954-967. doi:10.1016/j.neuroimage.2010.12.049
